# Ageing of inverse vulcanised polymers

**DOI:** 10.1039/d6lp00159a

**Published:** 2026-06-05

**Authors:** Christian W. Schmitt, Andy Mach, Valeria Berner, Eric Pohl, Joseph J. Dale, Birgit Huber, Dominik Voll, Emmanuel Richaud, Ute Schepers, Carl-Christoph Höhne, Patrick Théato

**Affiliations:** a Karlsruhe Institute of Technology, Soft Matter Synthesis Laboratory, Institute for Biological Interfaces III (IBG-3) Hermann-von-Helmholtz-Platz 1 76344 Eggenstein-Leopoldshafen Germany patrick.theato@kit.edu christian.schmitt3@kit.edu; b Fraunhofer Institute for Chemical Technology (ICT) Joseph-von-Fraunhofer Str. 7 76327 Pfinztal Germany; c Karlsruhe Institute of Technology, Institute for Functional Interfaces (IFG) Hermann-von-Helmholtz-Platz 1 76344 Eggenstein-Leopoldshafen Germany; d University of Vienna, Institute of Material Chemistry and Research Währinger Straße 42 Vienna 1090 Austria; e Karlsruhe Institute of Technology, Institute for Chemical Technology and Polymer Chemistry (ITCP) Kaiserstraße 12 76131 Karlsruhe Germany; f Laboratoire Pimm, Arts et Métiers Sciences et Technologies, CNRS CNAM Paris 75013 France

## Abstract

Inverse vulcanised polymers are an emerging class of materials with a broad range of applications from energy storage to fertiliser systems. As these materials are still subject of research, scaling to real-life applications is just underway. One major factor for these materials to be fit for industrial use is their ageing behaviour under environmental influences such as temperature, sunlight, moisture and pH or biological attack. Since ageing is rarely discussed in literature, we herein investigate the ageing behaviour of common inverse vulcanised polymers under real-life and simulated environmental influences. The results show strong structure-properties relationships depending on the comonomers used for the polymerisation. Aliphatic non-functional monomers produce rigid polymers with high resistance towards environmental influences, whereas bio-derived and functional monomers deliver more flexible materials that are prone to degradation by oxidation and hydrolysis. This study provides groundwork for future research into the design of sulfur polymers that require environmental stability or controlled degradation for their individual application.

## Abstract 2

The ageing behaviour of inverse vulcanised polymers, synthesised from fossil and renewable comonomers is investigated under thermal and radiative stress, aqueous environments and biological attack. Graphical abstract partly generated with NotebookLM.

## Graphical abstract

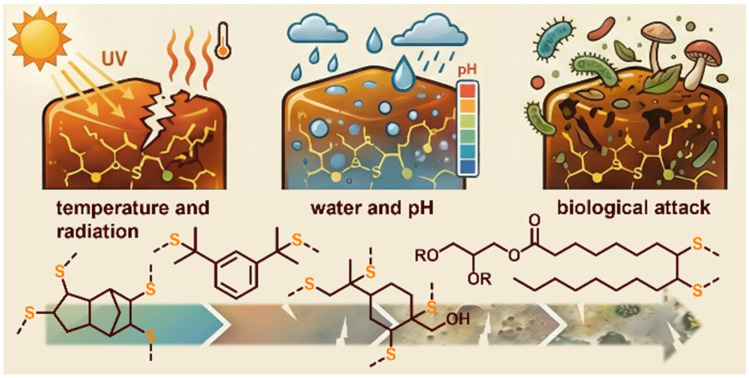

## Introduction

Among the most pressing environmental challenges today is the persistent pollution caused by hazardous chemicals released from *e.g.* plastic waste. These pollutants can remain in the environment for decades, posing serious threats to wildlife, eco-systems, and human health.^1^ Addressing this issue demands innovative approaches, including the development of environmentally friendly and more sustainable materials, one of which may be high-sulfur-content polymers.^2–6^ As with many advances in materials chemistry, their development has been driven by the valorisation of low-value industrial waste such as elemental sulfur, which is otherwise costly to store or dispose. Historically, the role of sulfur in polymer science has been largely limited to the vulcanisation of rubber, however, the development of inverse vulcanisation (IV) inverts this paradigm, using sulfur as a primary building block to synthesise entirely new classes of polymers.^7^ These materials often exhibit remarkable thermal, mechanical, and chemical properties, which can be finely tuned through adjustments in sulfur content and the choice of unsaturated crosslinker. Petrochemically derived comonomers such as dicyclopentadiene (DCPD), ethylidene norbornene, and 1,3-diisopropenylbenzene (DIB) typically yield polymers with higher glass transition temperatures (*T*_g_) and higher modulus. Other functional comonomers, such as siloxane derivatives or activated esters, allow precise tuning of glass transition temperatures.^8,9^ In contrast, renewable crosslinkers like myrcene, eugenol, and vegetable oils tend to produce more flexible materials with lower *T*_g_ values, but often with reduced shape persistency.^10–14^ This broad tunability has enabled IV polymers to be explored for a wide array of applications, including lithium–sulfur batteries,^15,16^ construction materials,^17–19^ antibacterial coatings,^20–24^ wastewater treatment and environmental remediation,^6,25–27^ infrared optics,^13,28,29^ and fertiliser delivery systems.^30–32^ Despite their versatility, the long-term stability and ageing behaviour of IV polymers remain poorly understood, largely due to the early stage of their development. As efforts to make use of these materials increase, a deeper understanding of their ageing mechanisms becomes increasingly important. The performance, reliability, and safety of IV polymers over time will ultimately determine their suitability for real-world applications. For example, a polymer used as an additive in Portland cement that degrades over time could compromise the structural integrity and lifespan of buildings. Similarly, a loss of antimicrobial efficacy due to leaching or oxidation of sulfur species would limit the utility of IV polymers in surface coatings or sprays designed to inhibit bacterial growth. In energy storage systems, instability under prolonged battery cycling could not only reduce performance but also pose safety hazards, given sulfur's flammability and its potential to release toxic gases at elevated temperatures.

In real-life applications, especially outdoors, many different environmental factors influence the ageing behaviour of polymeric materials, often with synergistic effects.^33–35^ The exposure to sunlight, and therefore ultraviolet light can induce bond scission and/or rearrangement, whereby the infrared spectrum in the sunlight is responsible for heating objects, which is even more prevalent for materials with darker colours.^36^ Excessive temperature variations can also break chemical bonds but, critically, imposes cyclic mechanical strain on materials due to expansion and contraction.^33^ Another major factor is water as it is ubiquitous in our environment, whether in the form of airborne humidity, rain or dew. Liquid water and water vapor can exert physical and chemical influences on more polar polymers, due to the capability of water to swell and leach components, as well as damage caused by hydrolytic reactions.^37,38^ With water also come microbiological attacks, where mould fungus growth is the most frequent cause of microbiological damage to materials predominantly used in the construction industry.^39,40^

**Fig. 1:**
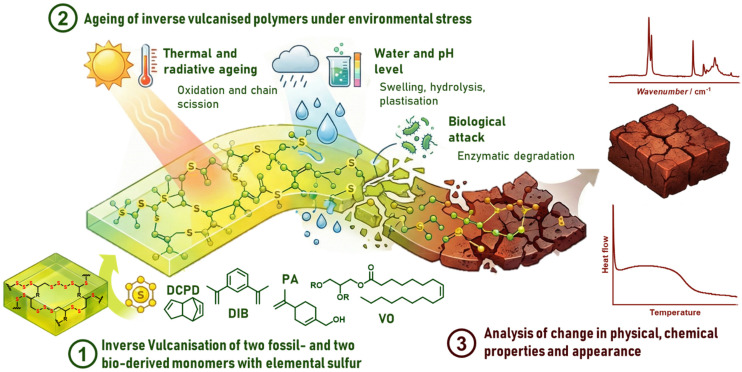
Schematic overview of the comonomers used for the inverse vulcanisation, applied environmental factors for ageing and investigated changes in properties.

Herein, we investigate the influence of environmental factors such as heat, radiation, water, and biological attack on inverse vulcanised polymers. Since their degradation behaviour is also highly dependent on the crosslinker structure, four common crosslinkers, namely DCPD, DIB, perillyl alcohol (PA) and vegetable oil (VO), more specifically sunflower oil, are used (Fig. 1).

## Results and discussion

### Synthesis of inverse vulcanised polymers

Four inverse vulcanised polymers were synthesised by the reaction of elemental sulfur with two fossil-based comonomers (DCPD, DIB) and two bio-based comonomers (PA, VO) in equal mass ratios (50/50, w/w) at 160 °C under stirring, with subsequent curing for 24 h at 140 °C, respective molar ratios of sulfur to C=C double bonds and detailed synthetic procedures are given in the SI. The resulting sulfur polymers are labelled p(50S-50DCPD), p(50S-50DIB), p(50S-50PA) and p(50S-50VO) with respect to the weight ratios of the individual components. The synthesis and subsequent ageing studies were conducted with consideration of the known sensitivity of high sulfur materials to sample history, particularly thermal exposure during synthesis and curing. In accordance with previous reports, indicating the influence of minor variations in thermal history on sulfur rich polymers, deviations in experimental values of glass transition temperatures are expected, even for formally identical samples.^41^

### Thermal ageing

Thermal ageing was performed at 150 °C in a ventilated drying oven under oxidative conditions (atmospheric air) for approximately 13 days to systematically investigate the degradation behaviour of the four inverse vulcanised polymers under accelerated ageing conditions. Further, this extreme ageing condition was chosen for all polymers to be above their glass transition temperature (see Fig. 3F), pushing it into over-curing state to force network degradation,^42^ also acting as a comparison to previous reports that investigated ageing under ambient conditions.^43^ Due to the high brittleness of p(50S-50DCPD), p(50S-50DIB) and p(50S-50PA) it was not possible to prepare sufficiently thin samples for thorough oxidation studies, therefore all samples were prepared with a thickness of approximately 2 mm for stability reasons. Samples for measurements were taken of their surface before and after thermal ageing since oxidation by oxygen diffusion is limited due to the samples thickness. In addition, the mass loss was recorded during the 13 days ageing period by weighing the remaining samples at specific time intervals.

**Fig. 2:**
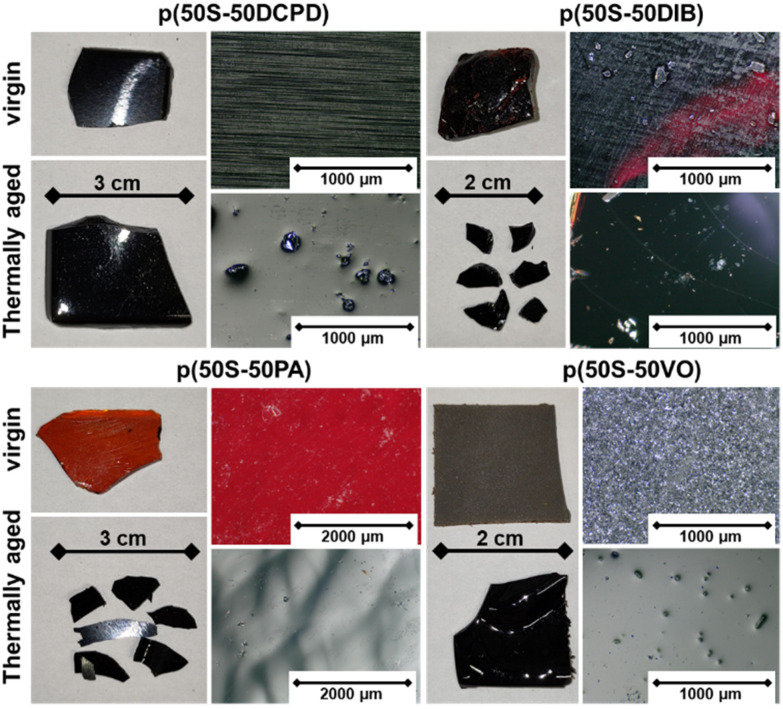
Images and micrographs of p(50S-50DCPD), p(50S-50DIB), p(50S-50PA) and p(50S-50VO) before and after thermal ageing for approximately 13 days at 150 °C.

**Fig. 3:**
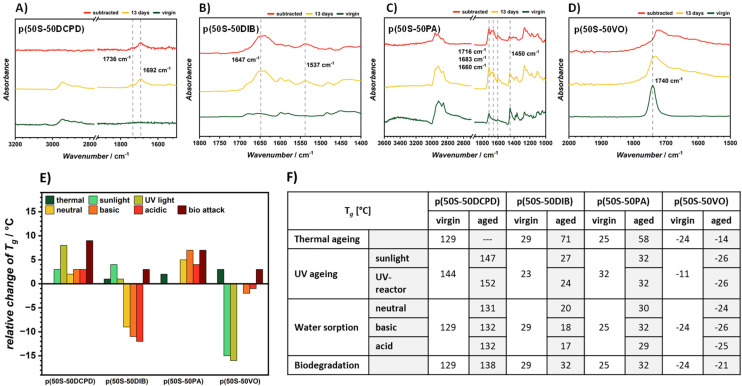
Zoomed in FTIR spectra of (A) p(50S-50DCPD), (B) p(50S-50DIB), (C) p(50S-50PA) and (D) p(50S-50VO) in the critical wavenumber regions before and after thermal ageing for approximately 13 days at 150 °C. Differential FTIR spectra are included for better visualisation of changes between virgin and aged polymers. For full spectra refer to Fig. S1 in the SI. (E) Relative change of the *T*_g_ to the virgin materials of all samples before and after exposure to thermal and radiative ageing, water sorption at various pH levels and biological attack. (F) Glass transition temperatures of p(50S-50DCPD), p(50S-50DIB), p(50S-50PA) and p(50S-50VO) before and after exposure to thermal and radiative ageing, water sorption at various pH levels and biological attack. For full thermograms refer to Fig. S2 in the SI. The *T*_g_ of the virgin materials differs due to batch-to-batch variations and possibly due to single DSC measurements, since results from different measurements can vary as well.

In the case of p(50S-50DCPD), there were no significant visual changes that could be observed between the virgin material and after thermal ageing for a prolonged time, as can be seen in Fig. 2. The respective micrographs however showed blisters in the size of approximately 100 µm, possibly due to structural rearrangement with increased chain mobility at elevated temperatures, or the release of occluded H_2_S gas. Further, the FTIR-ATR spectra revealed a pronounced increase in absorbance between 1770–1600 cm^−1^ (Fig. 3A), corresponding to the formation of carbonyl-containing oxidation products such as ketones and aldehydes. These species, which can also be indicative for an oxidative chain scission, are likely formed by thermally induced radical formation and subsequent reaction with atmospheric oxygen (proposed mechanisms are depicted in the SI).^44^ A concurrent decrease in the intensity of methyl (–CH_2_) stretching vibrations (3000–2800 cm^−1^) suggested degradation of aliphatic segments. In addition to these chemical changes, DSC analysis revealed a significant change in the *T*_g_, ranging from 144 °C to a non-measurable value (Fig. 3E and F). This observation was consistent with the high initial crosslink density and rigidity from the bicyclic structure of DCPD and further network densification by rearrangement of S–S bonds and thereby decrease in sulfur rank to a highly crosslinked polymer with high softening temperatures.^42,45^ A slightly increased onset temperature during TGA measurements, as well as minor mass loss of approximately 2% (Fig. S3 and S4) confirmed higher crosslink densities instead of evaporation of sulfur species. A summary of all TGA data (onset temperatures, char residue *etc*.) can be found in Table S1.

In contrast, p(50S-50DIB) exhibited a different response to thermal ageing. The colour changed from a dark, slightly transparent red to almost black (Fig. 2), and became more brittle as well, which was observed while removing samples from the oven when they easily broke by touch. FTIR-ATR spectra showed an increase in O–H stretching vibrations (3500–3100 cm^−1^), indicative of hydroperoxide, hydroxyl and carboxyl formation, along with intensified carbonyl absorbance in the 1700–1570 cm^−1^ region, suggesting oxidative cleavage of the polymer backbone (Fig. 3B, proposed mechanisms are depicted in the SI), especially due to the high reactivity of peroxides that can react with sulfur species to form alcohols and oxidised sulfur species.^46^ Notably, DSC measurements revealed a substantial increase in *T*_g_ from 23 °C to 71 °C (Fig. 3E and F), implying significant post-curing or additional crosslinking through dynamic sulfur bond rearrangement, explaining the increased brittleness. This behaviour was likely facilitated by the flexible structure of the DIB sections, which allowed for rearrangement of S–S bonds into shorter, more rigid linkages (decrease in sulfur rank), thereby enhancing the stiffness and thermal resistance of the polymer network.^43^ This was also implied by an increase in decomposition temperature (refer to Fig. S3 in the SI for TGA thermograms) and less mass loss in the first degradation step due to higher bond strength of C–S bonds compared to S–S bonds. Lower sulfur rank led to less S–S bonds in the material and therefore higher thermal stability and less mass loss.^45^ This is in agreement with literature findings, where sulfur containing polymers tend to form thermally stable char residue, especially with aromatic structures, comparable to the DIB comonomer.^47^ Increased carbon-bound regions in the FTIR spectra might also imply homo-polymerisation of DIB. Evaporation and further carbonation of the materials were also confirmed by the increased onset temperature and mass loss of ∼32% during prolonged thermal ageing as shown in Fig. S3 and S4. DIB is known to produce flexible, often linear polymers, with greater chain mobility than DCPD-derived sulfur polymers.^42^ We also suggest a structural rearrangement leading to aromatic stacking, and thus an increase in macrostructure ordering, increasing interchain interactions.^48^

The p(50S-50PA) material, synthesised using perillyl alcohol, demonstrated a different degradation behaviour compared to the two materials discussed above. Next to a colour change from orange to almost black upon thermal exposure (Fig. 2), FTIR-ATR spectra revealed a decrease in O–H stretching intensity (3570–3130 cm^−1^), consistent with the consumption of hydroxyl groups during oxidation (Fig. 3C, proposed mechanisms are depicted in the SI). Simultaneously, an increase in carbonyl absorbance (1750–1620 cm^−1^) was observed, indicating the formation of oxidation products such as aldehydes and carboxylic acid derivatives. DSC analysis showed a significant increase in *T*_g_ from 32 °C to 58 °C (Fig. 3F), suggesting denser crosslinking by decreasing sulfur rank. The presence of reactive hydroxyl groups in the perillyl alcohol moiety may have facilitated additional crosslink formation or promote the formation of shorter S–S bonds, contributing to the observed stiffening of the polymer matrix. In literature it is suggested, that cyclic alkenes are not as susceptible to side reactions involving sulfur compared to DIB or triglyceride derived inverse vulcanised polymers.^42^ Yet, the postulated mechanism for the oxidation of the polymer (evident from FTIR spectra) could promote the formation of hydrogen bonding and therefore, an increase in *T*_g_. Lastly, similar to p(50S-50DIB), the stiffening, colour and *T*_g_ change can also be explained by sulfur evaporation and carbonation with a concomitant highest mass loss among all samples of 39% after 13 days (Fig. S3 and S4).^47^ Further, mass loss during TGA measurements and increase of degradation temperature was not as pronounced compared to p(50S-50DIB), confirming less initial side reaction during inverse vulcanisation and higher sulfur ranks in cyclic alkenes.

A rather drastic change in properties was observed in p(50S-50VO), exhibiting significant colour change from brown to black, as well as a high brittleness compared to the flexible virgin material (Fig. 2). FTIR spectra revealed a broadened carbonyl region (1760–1500 cm^−1^), suggesting the generation of diverse oxidation products, including carboxylic acids, aldehydes, and ketones (Fig. 3D, proposed mechanisms are depicted in the SI). A decrease in –CH_2_ stretching vibrations (3000–2800 cm^−1^) further confirmed the oxidative degradation of aliphatic chains derived from the fatty acid components of vegetable oil.^42,49,50^ This also was evidence for carbonisation of the material over time, confirmed by TGA and mass loss of ∼30% during thermal ageing (Fig. S3 and S4). DSC measurements indicated an increase in *T*_g_ from −24 °C to −14 °C (Fig. 3F), reflecting an increase in crosslink density after thermal treatment. Interestingly, p(50S-50VO) exhibited the second lowest mass loss and a *T*_g_ shift of only 10 °C compared to all other samples and therefore surprisingly high thermal stability. Explanations are the formation of small molecular weight species and evaporation of sulfur due to bond breakage that can pack closer, therefore *T*_g_ increased, alongside higher brittleness due to chain–chain interactions and hydrogen bonding between oligomeric species. Mass loss from thermal ageing was also evidence for a carbonation mechanisms over time, next to depletion of subglassy relaxation, comparable to phenomena observed in epoxy resins (Fig. S3 and S4).^51^

In general, the change of *T*_g_ depends on the balance between chain scission and crosslinking, which during the ageing process develops with the change of sulfur ranks. Since molar ratios of sulfur to C=C bonds during synthesis was different for all samples, sulfur ranks and residual C=C bonds within the materials varied strongly. DIB for example was not capable of stabilising as much sulfur compared to DCPD, mostly due to the formation of linear structures, where double bonds only reacted on one position leading to higher sulfur ranks. Therefore, much of the sulfur was trapped as “dark sulfur”,^43,52^ which could act as additional crosslinker during prolonged thermal ageing, even though the ratio of sulfur to C=C was only approximately 2.5. Evaporation of this unreacted sulfur was also an explanation for the high mass losses at elevated temperatures. A similar trend was observed for p(50S-50PA). Inverse vulcanised vegetable oils on the other hand contain a large excess of sulfur over available C=C bonds with ratios of 6.9 to 9.4, depending on the fatty acids in the oil. This explained the strong recrystallisation of sulfur often observed in literature. Since vegetable oil can only stabilise a small fraction of the incorporated sulfur, with most of it recrystallising, the amount of sulfur available for further crosslinking and network densification during thermal ageing is limited, explaining the moderate increase in *T*_g_. Yet, the interpretation of thermally induced ageing effects must consider that chemical and physical changes may be spatially heterogeneous within the samples. Given the relatively large sample thicknesses, oxygen diffusion is expected to be very limited under the applied conditions, where oxidation is likely concentrated near the surface, while changes in thermal properties reflect responses of the material as a whole. Consequently, observations of oxidative signatures and concurrent changes in glass transition temperature or thermal stability are discussed as mechanistically consistent but not spatially equivalent processes.

### UV ageing

Inverse vulcanised polymers possess inherent susceptibility to ultraviolet (UV) irradiation due to the photochemical lability of polysulfide linkages, which might limit their long-term stability in outdoor environments without the use of UV stabilisers. However, sulfur compounds such as disulfides are used as secondary (preventive) antioxidants and UV stabilisers which act as hydroperoxide decomposers.^53^ Although inverse vulcanisation continues to gain attention for the development of more sustainable, sulfur-rich materials, the mechanistic understanding of their photo-degradation behaviour remains incomplete, particularly with respect to comonomer structure and exposure conditions. To elucidate these structure–property relationships, the four model systems were exposed to natural sunlight and to controlled UV-A irradiation in a laboratory reactor with a sample thickness of approximately 2 mm. Samples for analysis were taken of the surface, since UV irradiation is predominantly a surface effect, even though temperature fluctuations can alter the whole sample. The three-week sunlight ageing was performed in August 2025 over a period of 21 days, outdoors at Campus North of the KIT (zipcode: 76344, Germany, 49°5′39″N, 8°25′41″E) with temperatures varying from 34 °C to 5 °C and daily sunshine hours between 0 and 13 h (Fig. S5). Solar irradiation at ground level primarily comprised UV-A and a reduced UV-B component, while infrared radiation introduced thermal stress that can increase degradation. In contrast, artificial ageing in a home-built UV-reactor provided a uniform and intense UV-A flux (36 W at 310–400 nm, 350 nm peak) at constant 20 °C for 8 h per day (9:00–17:00 h) over a period of 21 days, allowing isolation of photo-chemical effects from thermal factors.

**Fig. 4:**
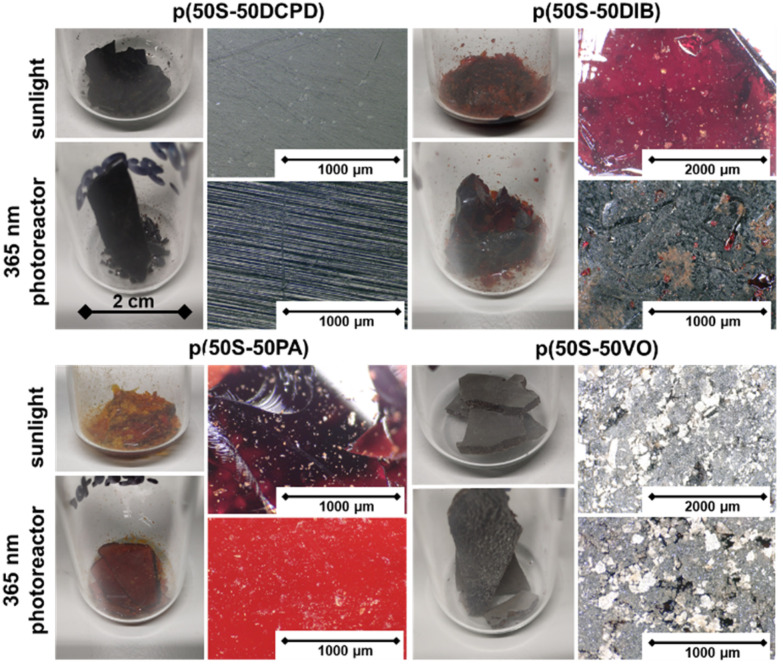
Images and respective micrographs of p(50S-50DCPD), p(50S-50DIB), p(50S-50PA) and p(50S-50VO) after exposure to sunlight and concomitant temperature variations, as well as UV light in a custom-built photo-reactor for 21 days. For images of the virgin material refer to Fig. 2.

**Fig. 5:**
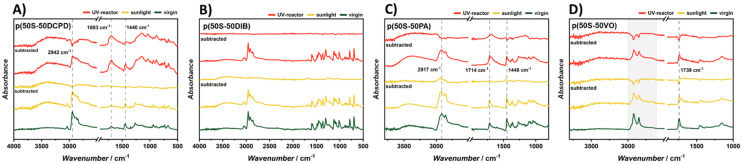
Zoomed in FTIR spectra of (A) p(50S-50DCPD), (B) p(50S-50DIB), (C) p(50S-50PA) and (D) p(50S-50VO) in the critical wavenumber regions after exposure to sunlight and concomitant temperature variations, as well as UV light in a custom-built photo-reactor for 21 days. Differential FTIR spectra are included for better visualisation of changes between virgin and aged polymers. For full spectra refer to Fig. S6 in the SI.

Among the tested materials, p(50S-50DCPD) revealed the highest photostability (Fig. 4), where FTIR spectra showed only moderate growth of hydroxyl and carbonyl species after irradiation, with more pronounced signatures under pure UV-A exposure (Fig. 5A). Interestingly, the appearance of a weak signal at 2661 cm^−1^ suggested the formation of thiols, which is a well-known mechanism for the homolytic oxidative cleavage of disulfides under UV-irradiation.^54,55^ Thiol formation was observed for all four samples, with more pronounced signals for p(50S-50DCPD) and p(50S-50DIB) which were more likely to have lower sulfur ranks compared to p(50S-50PA) and p(50S-50PA) as described in literature.^43,52^ Visually, there were no significant changes observed, even on the microscopic scale (Fig. 4). The stereo chemically constrained bicyclic DCPD units hindered radical mobility, spatially confining oxidation near the surface. DSC revealed a slight *T*_g_ increase from 144 °C to 147 °C under sunlight and a moderate increase to 152 °C in the photo-reactor, attributed to UV-activated sulfur–sulfur exchange reactions that enhance network densification (Fig. 3F), even though TGA thermograms showed no increase in thermal stability or solid residue after the ageing period (Fig. S9).

The DIB-based polymer displayed similar photo-oxidative resistance with only minor hydroxyl and carbonyl formation detected by FTIR, and increased oxidation under sunlight than under pure UV-A exposure (Fig. 5B). This inversed effect compared to DCPD highlights the accelerating role of solar heat in systems near their softening region. Indeed, p(50S-50DIB) possessed a *T*_g_ slightly above ambient temperature (approximately 23–24 °C, Fig. 3F), meaning sunlight exposure could heat the sample beyond *T*_g_, increasing chain mobility and facilitating photo-oxidation. *T*_g_ rose to 27 °C under sunlight but remained unchanged under UV-A exposure, consistent with localised network rearrangement only when thermal softening occurred. This demonstrates that this polymer was much more resilient towards thermal oxidation under regular environmental conditions compared to the accelerated approach discussed in the previous section.

Similarly, p(50S-50PA) exhibited modest photochemical sensitivity. Existing O–H groups complicated identification of freshly formed hydroperoxides, although UV-A irradiation produced detectable increases in hydroxyl and carbonyl absorbances due to selective oxidation (3500–3000 cm^−1^ and 1714 cm^−1^, Fig. 5C). Sunlight exposure induced far weaker chemical change (Fig. 3F), yet similar to the DIB derived polymer, p(50S-50PA) had a *T*_g_ close to ambient temperature (32 °C). Under strong sunshine, partial softening of the polymer matrix likely increased segmental motion and contributed to perceived surface melt behaviour, allowing degradation to progress to a limited extent even when UV effects alone were insufficient. As shown in Fig. 4 this resulted in loss of structural integrity of both materials p(50S-50DIB) and p(50S-50PA).

In contrast, the vegetable-oil-derived polymer p(50S-50VO) underwent extensive degradation under both sunlight and UV-A irradiation. FTIR confirmed substantial formation of hydroxyl and ester carbonyl groups (3500–3000 cm^−1^, 1750–1600 cm^−1^) consistent with severe oxidation of the organic backbone (Fig. 5D). Discoloration from brown-black to grey confirmed advanced deterioration in the UV-reactor compared to sunlight exposure (Fig. 4). On the microscopic scale, cracks in the surface was observed with darker areas and bright particles, which can be explained by recrystallisation of sulfur (sulfur blooming) and carbonisation of the glyceride backbone (dark areas).^56^ This was also in agreement with the FTIR spectra that show a decrease in –CH_2_ signals which was more pristine under strong UV light in the photo-reactor. DSC showed a *T*_g_ decrease from −10 °C to around −27 °C, reflecting chain scission and a loss of network integrity (Fig. 3F). TGA indicated major decrease in thermal stability, with onset decomposition temperatures dropping from 241 °C to 210 and 205 °C and lower early decomposition thresholds (Fig. S9). Sunlight ageing produced even poorer stability than pure UV-A exposure because thermal activation accelerated oxidative radical propagation within the highly susceptible aliphatic triglyceride-based matrix (Scheme S5).^50^ The increase of the first decomposition step in the thermogram (Fig. S9) also suggested the evolution of low molecular weight decomposition products as well as recrystallisation of elemental sulfur, also visible in the micrographs of the materials surface as mentioned earlier.

To put these observations into a broader context, it is important to note that UV-induced changes in inverse vulcanised polymers do not necessarily constitute purely destructive network degradation. Recent work by Chalker *et al.* has demonstrated that exposure to high-energy light can deliberately induce scission of polysulfide linkages, followed by recombination or network reorganisation, enabling spatially resolved degradation and reformation of polysulfides.^57^ Therefore, intense or short-wavelength irradiation may be regarded as a form of lithographic, highly accelerated ageing, in which photochemically activated S–S bond cleavage and exchange are driven far beyond the conditions encountered under natural sunlight. The comparatively mild chemical changes observed in the present study under UV-A irradiation (365 nm) and outdoor exposure, therefore reflects a different system dominated by superficial photo-oxidation and limited sulfur–sulfur exchange rather than an integral network rearrangement. Due to limited penetration depth of UV radiation and oxygen diffusion, photochemical modifications are likely confined to near surface regions, whereas changes in thermal properties reflect integrated responses of the polymer network. Bulk rearrangements observed after UV exposure may therefore arise indirectly from processes such as sulfur–sulfur exchange and localised heating, rather than uniform photochemical modification throughout the sample. This distinction highlights that IV polymers can display both apparent photostability under realistic environmental conditions and pronounced, stimulus-responsive behaviour under precise high-energy irradiation.

### Water uptake and hydrolysis

**Fig. 6:**
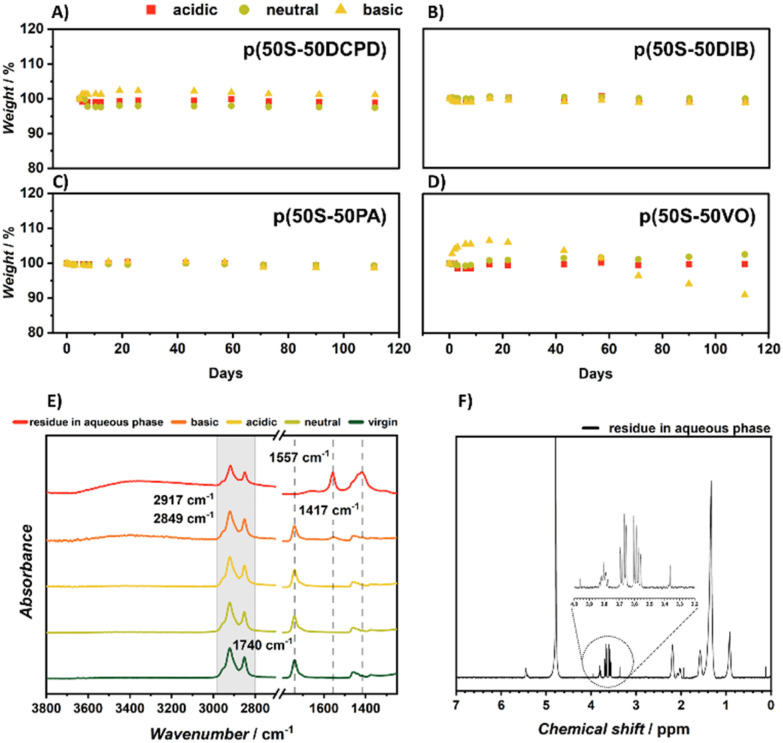
Relative weight variations of (A) p(50S-50DCPD), (B) p(50S-50DIB), (C) p(50S-50PA) and (D) p(50S-50VO) during the immersion in deionised water, 0.1 M NaOH and 0.1 M HCl solution for 110 days. (E) Zoomed in FTIR spectra of p(50S-50VO) before and after immersion in water with various pH levels as well as the residue in the aqueous phase after sorption under basic conditions. For full spectra of all samples refer to Fig. S10 in the SI. (F) ^1^H-NMR spectrum of the residue in the aqueous phase after sorption under basic conditions, recorded in D_2_O. The inlet is a zoom in the signals arising from the glycol derivatives.

Water uptake is another critical metric of materials, especially for building applications due to changes in weight and strength after water absorption and potential deterioration and failure resulting from freeze–thaw expansion and contraction cycles. Therefore, a systematic investigation was conducted on the respective sulfur polymer samples under acidic (0.1 M HCl), basic (0.1 M NaOH), and neutral (deionised water) conditions at ambient temperature with continuous monitoring of mass variation over a time period of 110 days with samples sizes of approximately 5 × 5 × 5 mm.

p(50S-50DCPD) demonstrated high resistance to water exposure. Across all aqueous environments, only minor mass gain or loss was observed (Fig. 6A), which can be attributed to superficial water adsorption under basic conditions, and leaching of unreacted species under neutral conditions, indicated negligible sorption or hydrolytic degradation. FTIR spectra remained unchanged relative to the virgin reference, confirming the chemical stability of the DCPD-based network (Fig. S10). DSC measurements revealed a slight increase in *T*_g_ from 129 °C to approximately 131–132 °C across all conditions, likely attributable to minor analytical variation (Fig. 3F). Overall, DCPD-based inverse vulcanisates largely retained their structural and thermal performance and exhibited uniformly excellent hydrolytic durability due to their high hydrophobicity and dense network structure that did not allow swelling.

The water exposure of p(50S-50DIB) resulted in no detectable changes in mass and therefore low sorption capability (Fig. 6B). However, FTIR detected a broad O–H absorption between 3660 cm^−1^ and 3100 cm^−1^ that evidenced physically adsorbed water on the materials surface and within (Fig. S10). This plasticising water infiltration caused substantial *T*_g_ changes due to increased chain mobility, lowering *T*_g_ values from 29 °C to 20 °C in neutral water, 18 °C in NaOH, and even further to 17 °C in acidic media (Fig. 3F). Thermal stability was moderately enhanced under neutral and basic conditions, as indicated by increased onset temperatures, while higher char yields aligned with the aromatic-rich structure supporting carbon residue formation (Fig. S13). Conversely, basic exposure reduced stability and lowered thermal onset and early decomposition thresholds, demonstrating that NaOH affected the sulfur-aromatic network more aggressively. The polymer maintained chemical integrity but softened slightly due to water uptake and/or NaOH-induced chain scission as already described in a different study (Scheme S6).^58^ Even though p(50S-50DIB) was a hydrophobic material, a small amount of absorbed water appeared to be sufficient to increase the chain mobility of the network, most likely due to a less dense structure compared to p(50S-50DCPD) especially since the initially suggested structure of highly crosslinked inverse vulcanised DIB was replaced by a linear structure providing more flexibility.^42^

The p(50S-50PA) polymer displayed good hydrolytic resistance, with no measurable mass variation in any solution (Fig. 6C), consistent with the absence of cleavable bonds. FTIR spectra before and after ageing were nearly identical, which indicated no chemical change within detectable limits (Fig. S10). *T*_g_ increased slightly for all conditions, reaching 30 °C under neutral exposure, 32 °C in NaOH, and 29 °C in acidic media, which can be explained by stronger hydrogen-bonding interactions with an increased moisture content (Fig. 3F). TGA showed stable thermal properties, with decomposition onset temperatures ranging from 204 °C to 215 °C which can be attributed to analytical variation.

A distinct degradation pathway occurred in p(50S-50VO), which contained hydrolysable ester groups. In neutral and acidic media, the polymer exhibited only slight weight increases due to water absorption, and FTIR spectra remained similar to the virgin material (Fig. 6D). *T*_g_ values of −25 °C (acidic) and −24 °C (neutral) closely matched the virgin *T*_g_ of −24 °C, indicating retention of molecular network flexibility and crosslink density (Fig. 3F). Yet, the respective TGA thermogram (Fig. S13) showed an increased char residue under neutral conditions, which can be explained by swelling and subsequent leaching of unreacted dark sulfur. However, the polymer exposed to NaOH differed fundamentally with a rapid initial mass increase of approximately 6%, confirming swelling as hydroxide-initiated reactions increased hydrophilicity due to the formation of carboxyl groups, followed by substantial mass loss of approximately 10% of initial weight. A pronounced yellow colouration in solution indicated progressive saponification and leaching of small molecules (Fig. S11, including proposed leached molecules in Scheme S7). FTIR data showed a broad O–H band between 3670 cm^−1^ and 3025 cm^−1^ and new carboxylate absorptions at 1610–1490 cm^−1^, together with reductions in ester C=O and C–O stretch intensities at 1740 cm^−1^ and 1160 cm^−1^, respectively (Fig. 6E). These findings confirmed ester cleavage, consistent with hydroxide attack resulting in alkoxide and carboxylate formation, followed by glycerol release as network connectivity failed. ^1^H NMR and FTIR analysis of the residue in the aqueous phase confirmed the formation of soluble glyceride containing degradation products (Fig. 6E and F). DSC showed a *T*_g_ shift to −26 °C due to chain scission and water-induced softening. The NaOH-driven degradation mechanism highlighted the specific vulnerability of triglyceride-derived comonomers under alkaline conditions, also evident from a decreased decomposition temperature (Fig. S13). Interestingly, neutral and acidic conditions increased the decomposition onset temperature. This can be explained by increased chain mobility due to plasticising effects of water and therefore facilitated network rearrangement and densification. Yet, more complex mechanisms also exist in the presence of moisture: prolonged exposure to moisture can also shift the equilibrium of S–S bond exchange, initiating a dynamic bonding structure and configurational rearrangement upon moisture sorption.^59–61^

### Biodegradation and toxicity

A simplified biodegradability experiment was conducted for 80 days, by placing the samples of p(50S-50DCPD), p(50S-50DIB), p(50S-50PA), and p(50S-50VO) in a compost container with household waste, provided from BVB biowaste digestion in Pfalz/Westheim (postal code: 67368, Germany), simulating natural degradation conditions. The compost was freed from impurities, not related to bio waste, including plastic and glass, before conducting biodegradability tests in a closed container on the terrain of Fraunhofer ICT in Pfinztal (postal code: 76327, Germany, 49°1′4″N, 8°31′14″E). The specific location allowed keeping track of meteorological data such as temperature and moisture to correlate the weather with potential structural changes within the polymer (Fig. S15).

**Fig. 7:**
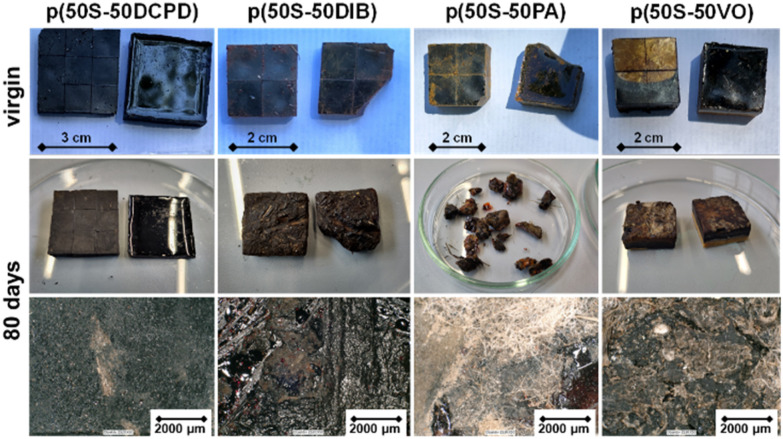
Images and respective micrographs of p(50S-50DCPD), p(50S-50DIB), p(50S-50PA) and p(50S-50VO) after exposure to biological attack, concomitant temperature and moisture variations in a household composter for 80 days.

**Fig. 8:**
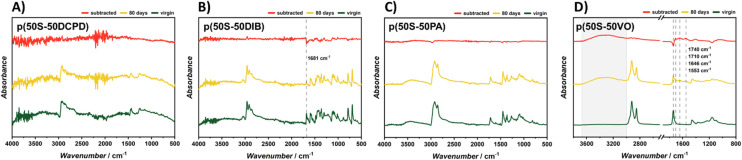
Zoomed in FTIR spectra of (A) p(50S-50DCPD), (B) p(50S-50DIB), (C) p(50S-50PA) and (D) p(50S-50VO) in the critical wavenumber regions after exposure to biological attack, concomitant temperature and moisture variations in a household composter for 80 days. Differential FTIR spectra are included for better visualisation of changes between virgin and aged polymers. For full spectra of all samples refer to Fig. S16 in the SI.

The experiment revealed distinct degradation and stability behaviours depending on the chemical structure of the comonomer. The inverse vulcanised polymer synthesised from DCPD exhibited no surface or morphological alterations after the composting test by the naked eye. On the microscopic level, the surface became more uneven and further compost residue stuck to the surface, which can be explained by superficial softening of the material during thermal peaks (Fig. 7). Yet, FTIR spectra of the virgin and aged specimens were identical, showing no new absorption bands or loss of characteristic peaks, which indicated chemical inertness toward microbial or oxidative attack (Fig. 8A). DSC analysis revealed a notable increase in *T*_g_ from 129 °C to 138 °C, corresponding to enhanced rigidity. This stiffening can be attributed to post-crosslinking reactions of residual unsaturated DCPD units and network rearrangement activated by the thermal conditions during composting (Fig. 3F). The absence of functional groups in DCPD, combined with its rigid hydrocarbon backbone, rendered the polymer essentially non-biodegradable under aerobic composting conditions. Instead, the moderate heat generated during microbial activity led to increased crosslinking density and stabilisation of the material.

p(50S-50DIB) showed major surface alterations following composting with the corresponding micrographs revealing imprints of compost residue, likely resulting from heat exposure rather than biological degradation due to the high temperatures during the composting process (∼68 °C) beyond its *T*_g_ (Fig. 7). FTIR spectra before and after ageing remained nearly identical, with no new signals of functional groups appearing and only a slight increase in the absorption band at 1680 cm^−1^ corresponding to the alkenyl C=C stretch of the aromatic comonomer (Fig. 8B), indicating the chemical preservation of the polymer network. DSC analysis revealed a small increase in *T*_g_ from 29 °C to 33 °C, signifying additional crosslinking of residual double bonds activated by thermal exposure above the polymer's *T*_g_ (Fig. 3F). TGA data confirmed improved stability with a higher decomposition onset from 217 °C to 227 °C (Fig. S18). The composting environment likely facilitated heat-induced polymer densification rather than biological attack. Overall, the DIB-based polymer maintained structural integrity, exhibited post-crosslinking effects, and showed no signs of microbial degradation, consistent with the absence of hydrolysable linkages or polar functional groups.

The p(50S-50PA) polymer underwent strong deformation, breakage and imprints of residue during composting, since the temperature inside the compost rose to approximately 68 °C, way above the *T*_g_ of the polymer (Fig. 7). Despite morphological changes, no chemical alterations were detected in FTIR spectra, which remained identical to those of the virgin polymer, indicating the absence of biodegradation or oxidation (Fig. 8C). The *T*_g_ increased from 25 °C to 32 °C, reflecting a more crosslinked or rigid network structure likely induced by heat-driven post-curing reactions (Fig. 3F). These observations demonstrate that p(50S-50PA) remained chemically intact during the composting process, with changes arising exclusively from exposure to elevated temperatures rather than microbial or enzymatic degradation. As both p(50S-50PA) and p(50S-50DIB) had glass transition temperatures slightly above ambient conditions, solar heating or exothermic microbial activity within the compost was sufficient to induce softening and shape deformation, but not chemical decomposition.

The vegetable oil-based inverse vulcanised polymer p(50S-50VO) exhibited the most pronounced evidence of partial biodegradation. Visual inspection revealed surface alterations, discoloration and cracking (Fig. 7), while FTIR spectra displayed new and intensified absorption bands consistent with oxidation and hydrolysis. A broad O–H stretching band between 3670 cm^−1^ and 3025 cm^−1^ indicated the formation of hydroxyl and carboxylic species, likely alcohols or carboxylic acids arising from microbial and chemical ester cleavage and absorbed moisture (Fig. 8D), confirming recent literature findings, showing that glycol containing derivatives form during decomposition of vegetable oil-based inverse vulcanisates.^62^ The C=O absorption near 1740 cm^−1^ became broader, suggesting oxidation to carbonyl-containing products such as aldehydes, ketones and carboxylates. Thermal analyses confirmed these observations: *T*_g_ increased slightly from −24 °C to −21 °C, likely due to partial post-crosslinking among sulfur chains and hydrogen bonding (Fig. 3F), while the onset of decomposition decreased from 252 °C to 235 °C (Fig. S18), which is an indication for loss of network integrity. This, on a first sight, contradictive results can be explained by oxidation of the organic components to acid groups leading to increase in the *T*_g_ due to H-bonding (further evident from broadening on the FTIR bands) while structural breakdown occurred, decreasing decomposition temperature.

Lastly it has to be considered, that under the present ageing conditions, combining thermal stress, moisture and biological attack, surface level chemical interactions occur alongside bulk effects throughout the whole sample. As a result, network rearrangement and post curing effects may occur independently of biological activity, complicating direct attribution of bulk property changes to biodegradation alone. Where surface chemical modification is observed, these effects are considered spatially confined and not necessarily representative of uniform degradation throughout the material. The combined analytical response therefore reflects a superposition of surface initiated chemical processes and bulk averaged thermal history effects.

**Fig. 9:**
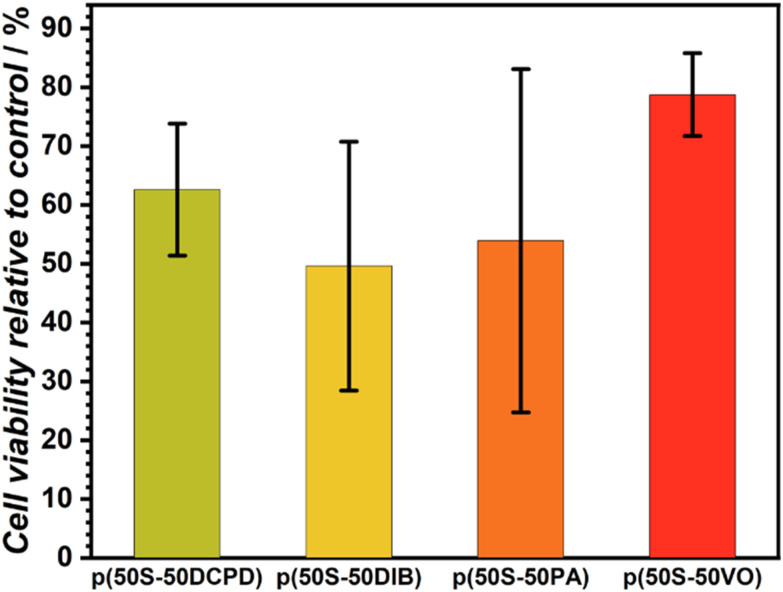
Cell viability (in % relative to control experiment) after treatment with ground sulfur polymers (p(50S-50DCPD), p(50S-50DIB), p(50S-50PA), and p(50S-50VO)).

In addition, a 3-(4,5-dimethylthiazol-2-yl)-2,5-diphenyl-tetrazoliumbromid (MTT) assay was employed as a viability test to evaluate the material's impact on cellular health. MTT, a water-soluble tetrazolium salt, reduced by metabolic active cells to formazan, an insoluble compound. This transformation results in a colour change from yellow to blue-purple, which can be quantitatively assessed using a photometer at a wavelength of 595 nm. This method provides a quantitative measurement of cell viability and is used in this study to gain first insights on the environmental toxicity of certain inverse vulcanised polymers. The assay was performed by grinding the polymers into powder and dispersing it in cell media at 10 mg ml^−1^. The dispersion was added to HeLa cells at a density of 1 × 10^5^ cells per well and incubated for 72 h before MTT addition. It is known that inverse vulcanised polymers have inherent antimicrobial properties, which might enhance cytotoxic effects and further raising the question whether mammalian cells would show a similar response.^21,24,63^ Yet, studies on the cytotoxicity on high sulfur polymers with bio-based comonomers showed low to non-toxic properties, but it was suggested that different comonomers and material dimension (bulk material, nanoparticle, coatings *etc*.) might change their toxicity. A similar trend could be observed in this study, where p(50S-50VO) showed the highest cell viability of approximately 80%, when all other polymers exhibited moderate cell viability between 50 to 65% (Fig. 9). Minor amounts of unreacted monomer or polymer fragments can be an explanation. However, as a general observation, bio-based comonomers appeared promising for applications where cytocompatibility is relevant. Although p(50S-50PA) showed only moderate mean viability, the spread across replicates overlapped with literature values for comparable bio-based systems.^21^ The MTT assay was performed at 37 °C, which was above the *T*_g_ of p(50S-50DIB) and p(50S-50PA) but below that of p(50S-50DCPD). As increased sulfur mobility above *T*_g_ has been linked to enhanced antibacterial activity,^21^ we hypothesise that the same mechanism may contribute to the lower viability observed for the low-*T*_g_ formulations, while the more rigid p(50S-50DCPD) matrix restricts sulfur leaching and thus preserves cell viability.

## Conclusions

The major takeaway message of this study is that comonomer structure is a key determinant of stability and degradation pathways in inverse vulcanised polymers across thermal, photo-oxidative, hydrolytic, and biological environments. Yet, for the herein proposed mechanisms, such as network compaction through sulfur rank reduction, sulfur species evaporation, dark sulfur participation, thiol formation and aromatic stacking, additional studies are required to confirm the involved mechanisms. Rigid, non-hydrolysable monomers such as DCPD and DIB in most aspects consistently generate networks with exceptional chemical, thermal, and UV stability, making them strong candidates for long-lived materials in harsh or outdoor applications, even though their high brittleness might be a drawback. PA offers intermediate adaptability: while structurally stable, its mobility near *T*_g_ enabled controlled softening or reconfiguration under elevated temperatures. In contrast, VO introduced degradable ester functionalities, enabling partial biodegradation as well as low cytotoxicity, but at the expense of thermal and environmental stability. Importantly, while quantitative values may vary, the qualitative ageing trends and structure–property relationships discussed herein were consistent across repeat experiments, highlighting the intrinsic sensitivity of inverse vulcanised polymers to processing history and the need for cautious comparison between studies.

These insights revealed a powerful structure–property toolbox for inverse vulcanised materials: rigid and aromatic comonomers confer durability, while aliphatic ester-containing monomers promote degradability. By strategically selecting or blending comonomers, sulfur-rich polymers can be designed with tuneable lifetimes, ranging from persistent engineering materials to environmentally degradable systems. Moreover, functional groups within the polymer structure provide possibilities for further functionalisation and controlled degradation, but also pose risks for oxidation and uncontrolled decomposition thereby which has to be accounted for when designing these materials. Additional research should focus on the development of suitable additives for sulfur rich polymers to further enhance their stability and broaden the range of applications. The ageing framework presented herein provides a foundation for future development of sulfur-based polymers optimised for long-term performance, recyclability, and effective end-of-life treatment.

## Author contributions

C. W. S.: conceptualisation, data curation, formal analysis, investigation, project administration, supervision, visualisation, validation, writing – original draft, A. M.: data curation, formal analysis, investigation, writing – original draft, V. B.: formal analysis, investigation, supervision, validation, writing – review & editing. E. P.: data curation, formal analysis, investigation, validation, writing – original draft, J. J. D.: data curation, writing – review & editing, B. H.: investigation, D. V.: supervision, validation, writing – review & editing, E. R: supervision, validation, writing – review & editing, U. S.: resources, supervision, writing – review & editing, C.-C. H.: supervision, validation, writing – review & editing, P. T.: funding acquisition, project administration, supervision, writing – review & editing.

## Conflicts of interest

There are no conflicts to declare.

## Supplementary Material

LP-004-D6LP00159A-s001

## Data Availability

The data supporting this article have been included as part of the supplementary information (SI). Supplementary information is available. See DOI: https://doi.org/10.1039/d6lp00159a. Original data for this article, including ATR-FTIR, DSC, NMR, TGA, micrographs and cytotoxicity assays are available at Radar4Chem at https://doi.org/10.22000/45q6jyng7cnmqku3.
