# Supplementary material for: Ageing of inverse vulcanised polymers
Source: RSC Appl Polym. 2026 Jun 5;4(4):1196–207. doi: 10.1039/d6lp00159a (PMC13270288; doi:10.1039/d6lp00159a)
Supplement: LP-004-D6LP00159A-s001 [file LP-004-D6LP00159A-s001.pdf]

Supplementary Information (SI) for RSC Applied Polymers.
This journal is © The Royal Society of Chemistry 2026

Supporting Information

Ageing of Inverse Vulcanised Polymers

Christian W. Schmitt,[a#*] Andy Mach,[#] Valeria Berner,[b] Eric Pohl,[c] Joseph J. Dale,[d] Birgit Huber,[a] Dominik Voll,[e] Emmanuel Richaud,[f] Ute Schepers,[c] Carl-Christoph Höhne,[b] Patrick Théato[a,e*]

[a] P. Théato, C. W. Schmitt, B. Huber
Karlsruhe Institute of Technology
Soft Matter Synthesis Laboratory
Institute for Biological Interfaces III (IBG-3)
Hermann-von-Helmholtz-Platz 1, 76344 Eggenstein-Leopoldshafen, Germany
E-mail: patrick.theato@kit.edu, christian.schmitt3@kit.edu

[b] C.-C. Höhne, V. Berner
Fraunhofer Institute for Chemical Technology ICT
Joseph-von-Fraunhofer Str. 7, 76327 Pfinztal, Germany

[c] U. Schepers, E. Pohl
Karlsruhe Institute of Technology
Institute for Functional Interfaces (IFG)
Hermann-von-Helmholtz-Platz 1, 76344 Eggenstein-Leopoldshafen, Germany

[d] J. J. Dale
Institute of Material Chemistry and Research
Faculty of Chemistry
University of Vienna
Währinger Strasse 42, Vienna 1090, Austria

[e] P. Théato, D. Voll
Karlsruhe Institute of Technology
Institute for Chemical Technology and Polymer Chemistry (ITCP)
Kaiserstraße 12, 76131 Karlsruhe, Germany

[f] E. Richaud
Laboratoire Pimm
Arts et Métiers Sciences et Technologies
CNRS CNAM, Paris 75013, France

Contents

AI Tool usage statement 3
Materials 3
Methods 3
  Thermogravimetric analysis (TGA) 3
  Differential scanning calorimetry (DSC) 3
  Nuclear Magnetic Resonance Spectroscopy (NMR) 3
  Attenuated total reflecting Fourier transform infrared spectroscopy (ATR-FTIR) 4
  Photometer 4
  Synthesis of inverse vulcanised polymers 4
  Ageing tests 4
    Thermal ageing 4

UV ageing 5
Water sorption 5
Biodegradation 5
Cytotoxicity test 5
Supplementary data 6
Thermal ageing 6
UV ageing 9
Water sorption 12
Biodegradation and cytotoxicity 14
TGA data summary 18
Proposed ageing mechanics 19
Reduction of sulfur rank 19
Oxidation 19
p(50S-50DCPD) 19
p(50S-50DIB) 19
p(50S-50PA) 20
p(50S-50VO) 20
Hydrolysis 20
References 21

AI Tool usage statement

For language polishing and editing, Microsoft Copilot was used under corporate data safety licence.

Parts of the TOC figure and parts of figure 1 were generated by Google NotebookLM.

Materials

Elemental sulfur (Thermo Scientific), perillyl alcohol (BLDpharm), 1,3-diisopropenylbenzene (TCI), dicyclopentadiene (Sigma-Aldrich), sunflower oil was purchased at REWE Germany.

Methods

Thermogravimetric analysis (TGA)

TGA measurements were performed at ENSAM using a TA instrument of the Q500 series before and after the alteration process. Under nitrogen with a flow rate of 60 mL min^{-1}, 5-20 mg of previously dried sample mass was placed in a platinum pan and heated continuously at 10 °C min^{-1} rate up to 700 °C. After reaching the temperature, the RAMP proceeded into holding the temperature under isothermal conditions for at least 50 min.

At Karlsruhe Institute of Technology measurements were performed using a TGA 5500 from TA instrument under nitrogen flow. 5-10 mg of previously dried sample mass was heated at 10 °C min^{-1} rate up to 700 °C before holding the temperature under isothermal conditions for at least 5 minutes.

For all TGA measurements the onset temperature was determined using the tangent approach: thereby the tangent intersection of baseline and at maximum slope were used to obtain the onset temperature of the corresponding decomposition process.

Differential scanning calorimetry (DSC)

At ENSAM, a differential scanning calorimeter from TA instrument, DSC 25 was used to obtain the glass transition temperature (T_g) via the tangent approach. For the measurement up to 10 mg of sample mass was placed in an aluminium crucible at a temperature between 0-200 °C. This involves a heating rate of 10 °C min-1 under inert conditions (N_2). Only one measurement per sample was conducted. Values for the T_g were determined from the 2nd heating ramp.

DSC measurements at Campus North (KIT) at SML using a DSC 2500 from TA instruments under nitrogen flow. Hermetic aluminium pans with an additional hole in the lid were used to identify the glass transition temperature (T_g) of cured samples at a temperature range from -40 °C to a maximum of 250 °C with a heating rate of 10 °C min^{-1}. The values for the T_g were also obtained from the 2nd heating cycle by using the tangent method.

Nuclear Magnetic Resonance Spectroscopy (NMR)

The NMR spectra of synthesised flame retardant agents were recorded on Bruker Avance 400 NMR spectrometer at 400 MHz for ^{1}H-NMR. Chloroform-d_1 (produced from the company Eurisotop) and Water-d_2 (from Carl Roth) were deployed as deuterated solvent. To evaluate the spectra, $CDCl_3$ (7.260 ppm, s for ^{1}H-NMR and D_2O (4.790 ppm, s for ^{1}H-NMR) were used as residual solvent signals.

Attenuated total reflecting Fourier transform infrared spectroscopy (ATR-FTIR)

FTIR-ATR measurements were performed on a Bruker Alpha II ATR-IR spectrometer from 400 cm^{-1} to 4000 cm^{-1} with a resolution of 4 cm^{-1} and 24 scans.

Differential FTIR spectra were obtained by normalising the reference spectra (virgin IV polymer) and the sample spectra (aged IV polymer), then subtracting the reference spectrum, multiplied by a scale factor, from the sample spectrum: (Sample) – (scale factor)(reference) = differential spectrum.
the scale factor was set by iterative adjustment until the chosen reference peaks were flat with the baseline.

Photometer

The SpectraMax® iD3 Multi-Mode Microplate Reader from Molecular Devices was utilised to measure the absorbance of MTT 96 well plates. The measurement of these plates was conducted at a wavelength of 595 nm at room temperature.

Synthesis of inverse vulcanised polymers

To obtain inverse vulcanised polymers, molten sulfur was converted under dropwise addition of an unsaturated comonomer. Starting off, by melting elemental sulfur at 140 °C in a big crimp vial, various kinds of comonomers were inserted ranging both in rigidity and structural flexibility. For the sulfur crosslink, 50% comonomer by mass was used (sunflower oil, Dicyclopentadiene (DCPD), 1,3-Diisopropenylbenzene (DIB) and perillyl alcohol).

Each comonomer was added to the molten sulfur at 140 °C under constant stirring. For some IV polymers the temperature was gradually increased to a specific temperature and stirred for an exact time were the reaction mixture is still liquid so it can be poured into moulds. The detailed reaction conditions for every IV polymer can be seen in the table below. Upon completion, the reaction mixture was poured in a mould and cured in an oven for 24 h at 140 °C.

Sample	Comonomer wt.%	Sulfur wt.%	Molar ratio mol(S):mol(C=C)	Molar ratio mol(S_8):mol(C=C)	T stirred °C	Duration min
**p(50S-50VO)**	50	50	6.89-9.39	0.86-1.18	150	25
**p(50S-50DCPD)**	50	50	2.06	0.26	140	70
**p(50S-50PA)**	50	50	2.38	0.3	160	110
**p(50S-50DIB)**	50	50	2.46	0.31	160	30

Ageing tests

Thermal ageing

For the thermal aging process, certain conditions had to be defined in order to ensure results that are as reproducible as possible. One of them refers to sample thickness as external stimuli induced surface alteration can be captured via FTIR spectra before and after the alteration. However, for thicker samples, the homogenous aging throughout the whole material cannot be guaranteed and may lead to different test results when relying on instruments such as TGA or DSC. The thermal stability monitored by TGA as well as the glass transition temperature (T_g) measured by DSC would vary depending on the oxidised state of the aged sample.

Thermal aging was initiated by placing cured IV samples in an oven at 150 °C. The alteration process was monitored in via FTIR-ATR, DSC and TGA.

UV ageing

UV aging of IV polymers was performed under sunlight as well as in a UV reactor. For aging under sunlight, IV polymers were placed in 20 mL vials, all assembled in a test tube stand and attached outside building 319 at Campus North, Karlsruhe Institute of Technology. The artificial light exposure was carried out in a custom-made UV reactor with an ultraviolet lamp from Philips Cleo Compact PL-L (5 × 36 W), covering a wavelength area from 310 – 400 nm with the highest intensity at 350 nm.

Both of the experiments were conducted from the 11th August until the 1st September within a three-week time frame to investigate potential changes induced by UV light. Whilst the natural UV aging was carried out throughout the whole period, the aging in a UV reactor was only conducted at a specific time between 9 am till 5 pm.

The analytical data was obtained via DSC, TGA and FTIR before and after the alteration process.

Water sorption

Water sorption of IV-polymers was investigated by placing cured IV samples in 50 mL centrifuge tubes under basic, neutral and acidic conditions, respectively at ambient temperature. Each sample was filled with 20 mL 0.1 M NaOH, 0.1 M HCl or deionised water and weighed accordingly over a defined time period. Starting off with short time intervals which then progressed into longer time frames.

Samples with high water sorption potency were dried before they were subjected to TGA, DSC, and FTIR-ATR measurements.

Biodegradation

A simplified biodegradability test was executed from 11th June till 1st of September, by placing cured IV samples in a compost container with biological waste provided from BVB biowaste digestion in Pfalz/Westheim (postal code, 67368). The compost was freed from impurities, not related to bio waste including plastic and glass before conducting biodegradability tests in closed containers on the terrain of Fraunhofer ICT in Pfinztal. The specific location allows keeping track of meteorological data to correlate the weather with potential structural changes within the polymer, measured via DSC, TGA and IR before and after the experiment. Moisture content was measured by taking a sample of the composting material, drying it and measuring its weight before and after.

Specific parameters were monitored during the biodegradability experiment such as temperature and compost moisture content.

Cytotoxicity test

All work is carried out under the sterile bench. The cells are first thawed and seeded into a cell culture flask covered with DMEM containing 10% FBS, 1% L-alanyl-L-glutamine and 1% penicillin-streptomycin. The cells are then incubated at 37 °C in 5% CO_2. For use, 20 mL of a cell concentration of 105 cells/mL must first be prepared. First, the cell culture flask is examined under the microscope for impurities and contamination. If none are found, the cell culture medium is aspirated from under the safety bench and 10 mL of sterile PBS is added to the flask to remove the remaining cell culture medium. This wash solution is also aspirated. Then 2 mL of trypsin/EDTA is added to the cells and the bottle is incubated at 37 °C for 4 min. At the end of the incubation period, the cells are dislodged from the bottom of the

bottle by gently tapping. The dissolution of the cells can be observed under a microscope. Instead of adhering flat to the bottom, small round cells can be seen floating in the solution. 6 mL of cell culture medium is added to the bottle again under the safety bench to inactivate the trypsin. The cells are then transferred to a centrifuge tube and centrifuged at 1400 rpm for 6 min. After 6 min, the supernatant is discarded. Some liquid is retained to re-suspend the cell pellet. Subsequently, 10 mL of fresh cell culture medium is added to the tube. The cells are enumerated using the Neubauer chamber to calculate the cell concentration of the existing cell suspension and to prepare a cell suspension with a cell concentration of 105 cells/mL. The calculated volume of the cell suspension is then transferred to a tube and filled to a volume of 10 mL with cell culture medium. The MTT assay is performed to determine the cytotoxicity of the sulfur polymers. It is performed under sterile conditions to avoid contamination of the cell culture. All liquids utilised for the preparation or dilution of the solution must be sterile. The experiment was performed with fibroblasts to investigate possible toxicity on the skin. The cell suspension, which had been prepared in advance, was transferred to a plastic tube. Then, 100 µL of the cell suspension was seeded in a 96-well plate using a multichannel pipette. The edge of the well plate is not covered. The plates are then incubated for 24 hours at 37 °C and 5% CO_2.

Supplementary data

Thermal ageing

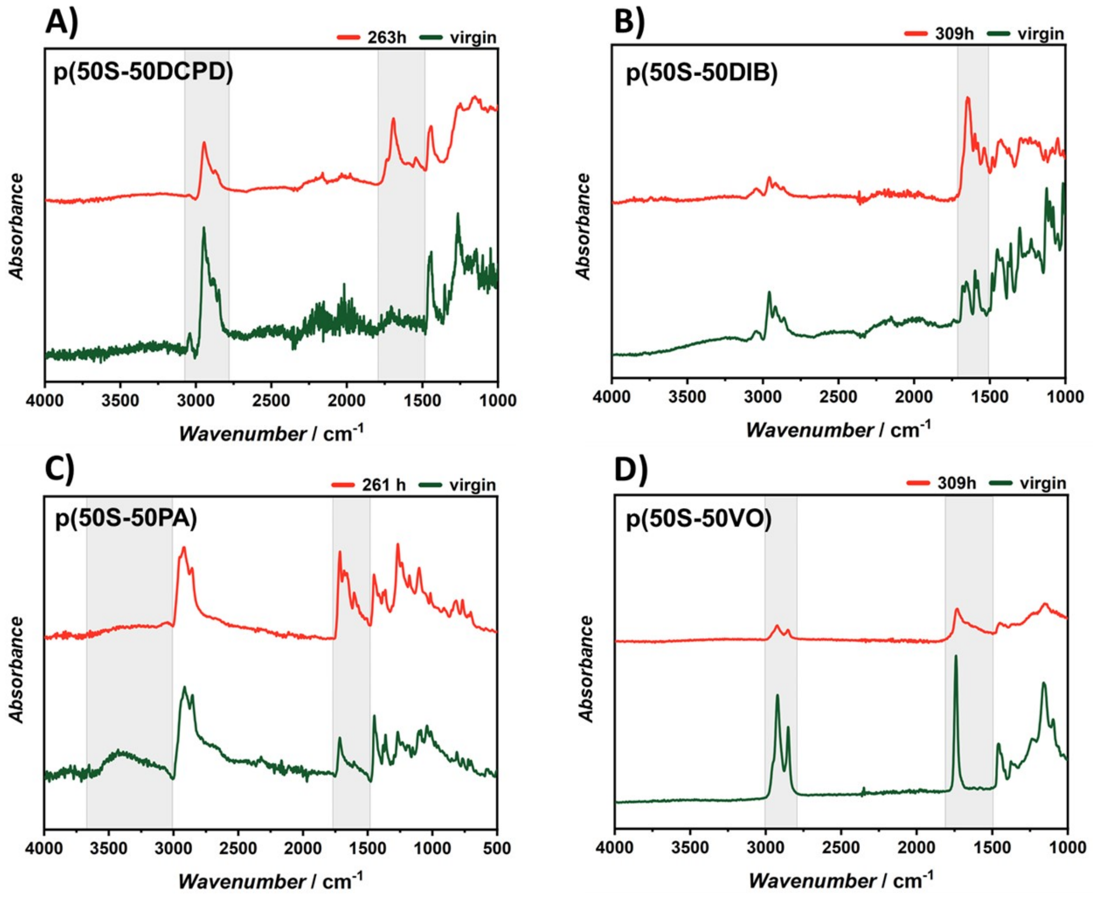

Figure S 1: FTIR spectra of p(50S-50DCPD), p(50S-50DIB), p(50S-50PA) and p(50S-50VO) before and after ageing at 150 °C for approximately 13 days.

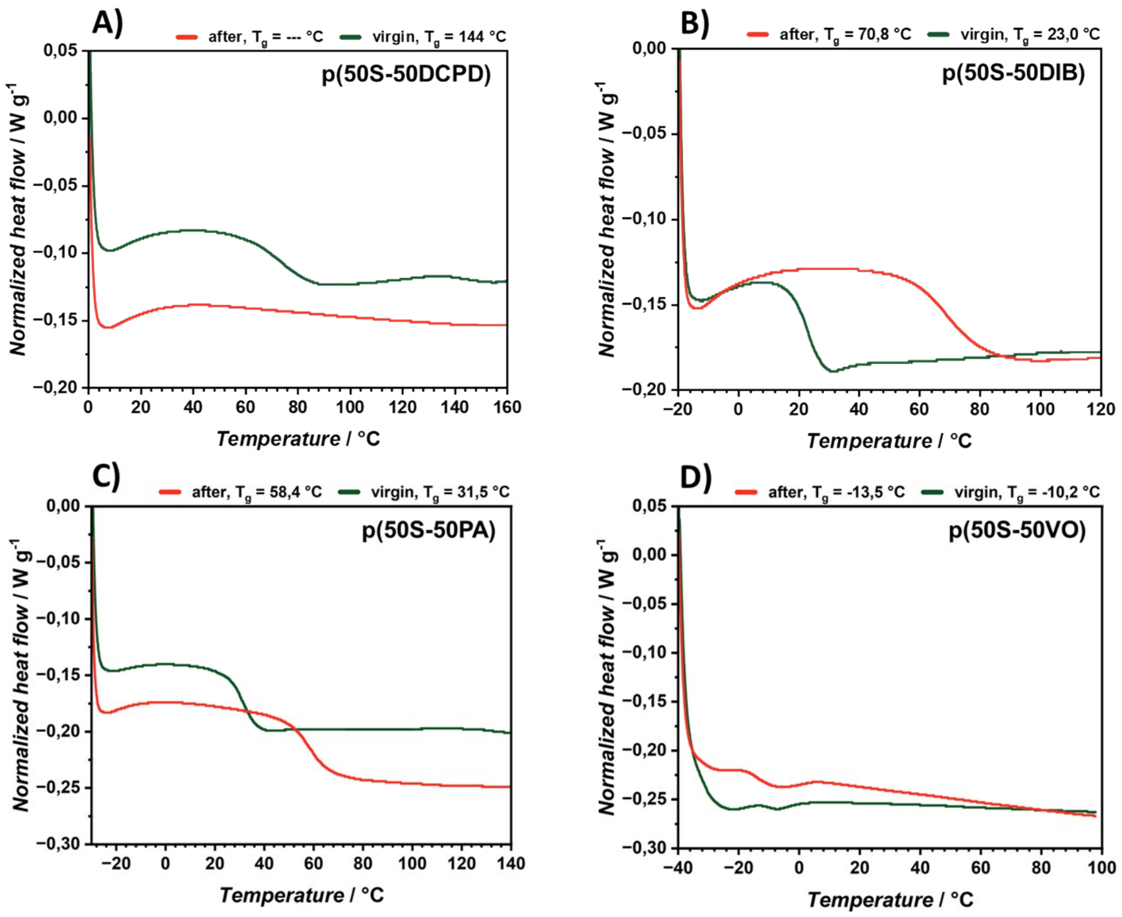

Figure S 2: DSC thermograms of p(50S-50DCPD), p(50S-50DIB), p(50S-50PA) and p(50S-50VO) before and after thermal ageing at 150 °C for 13 days.

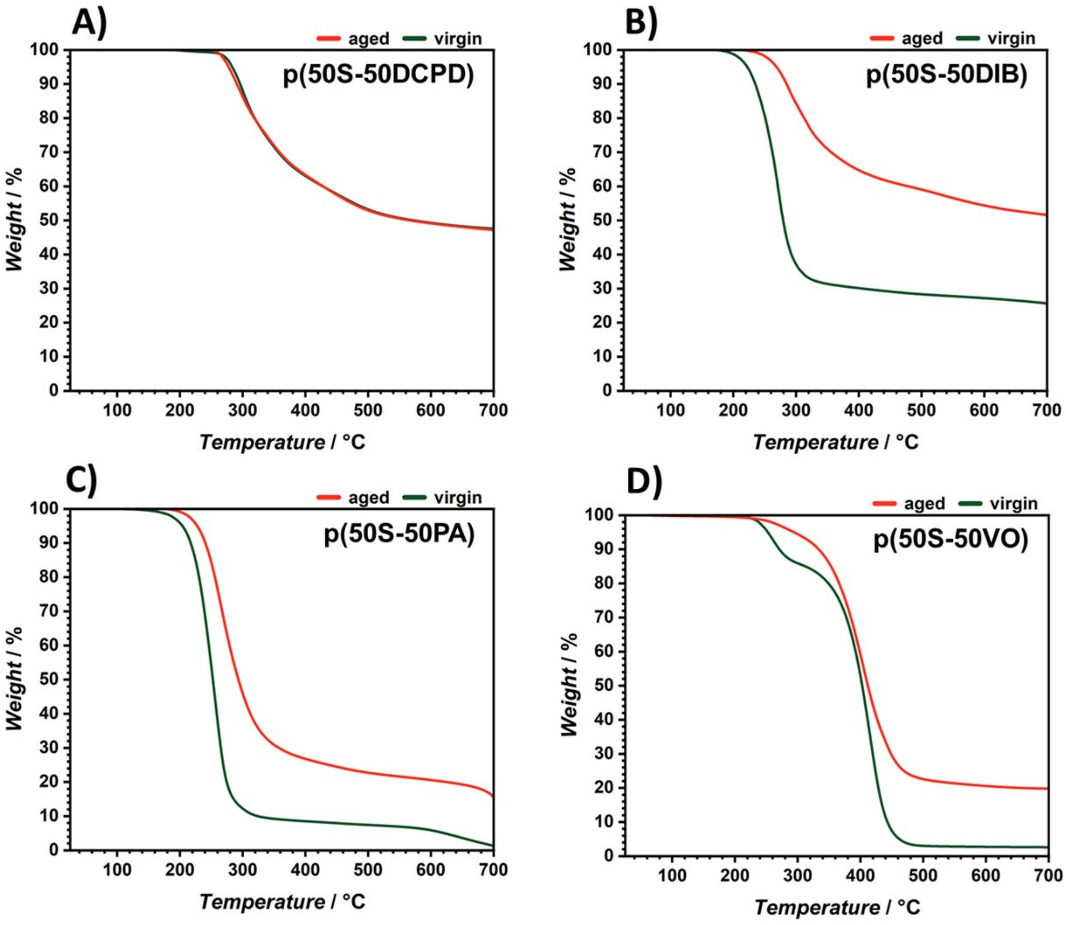

Figure S 3: TGA thermogram of p(50S-50DCPD), p(50S-50DIB), p(50S-50PA) and p(50S-50VO) before and after exposure to thermal ageing at 150 °C for 13 days.

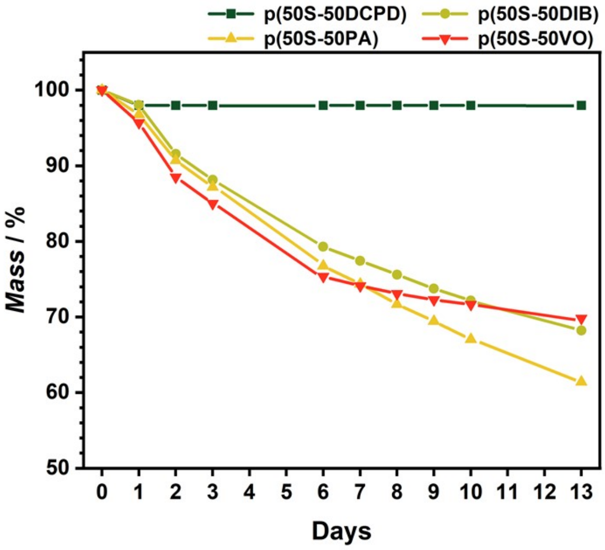

Figure S 4: Mass loss of p(50S-50DCPD), p(50S-50DIB), p(50S-50PA) and p(50S-50VO) during exposure to thermal ageing at 150 °C for 13 days.

UV ageing

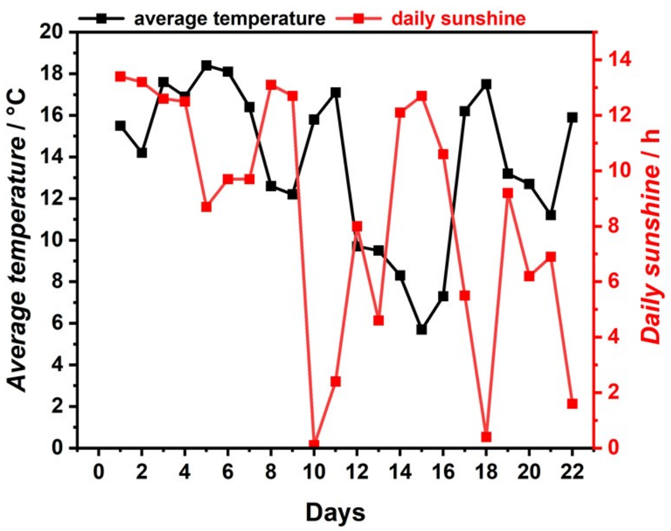

Figure S 5: Average daily temperature and hours of sunshine during the UV ageing test.

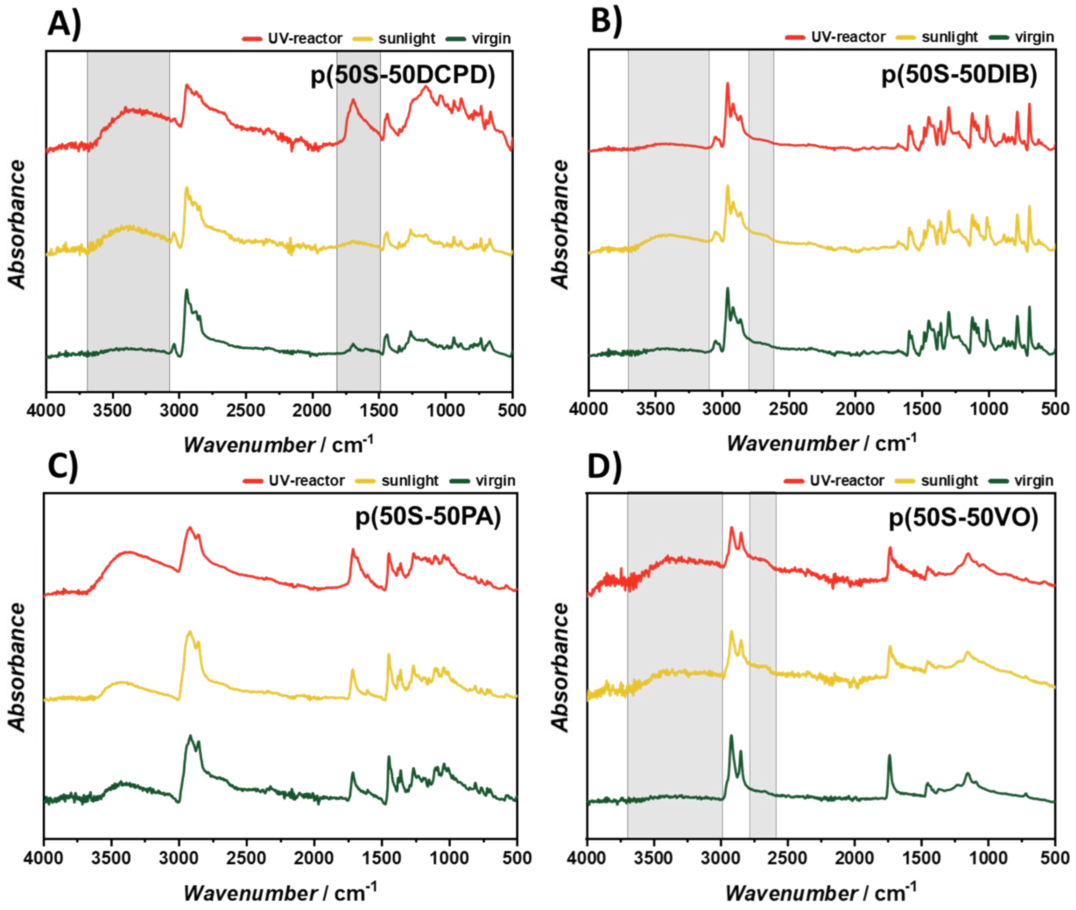

Figure S 6: FTIR spectra of p(50S-50DCPD), p(50S-50DIB), p(50S-50PA) and p(50S-50VO) before and after exposure to sunlight and concomitant temperature variations, as well as UV light (365 nm) in the custom-made photo-reactor.

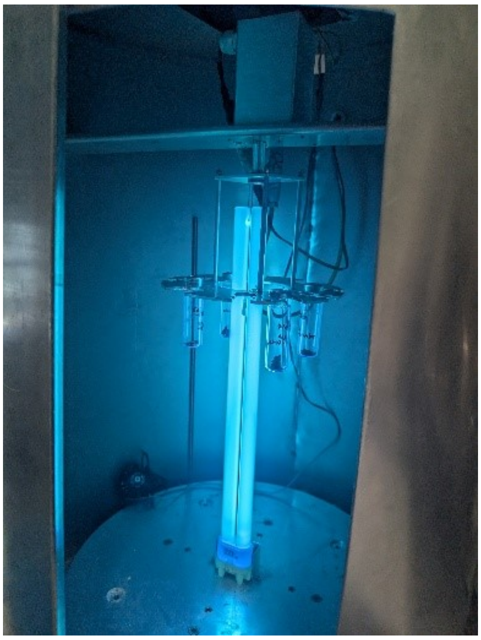

Figure S 7: Image of the custom-made photo-reactor equipped with a 365 nm UV-lamp.

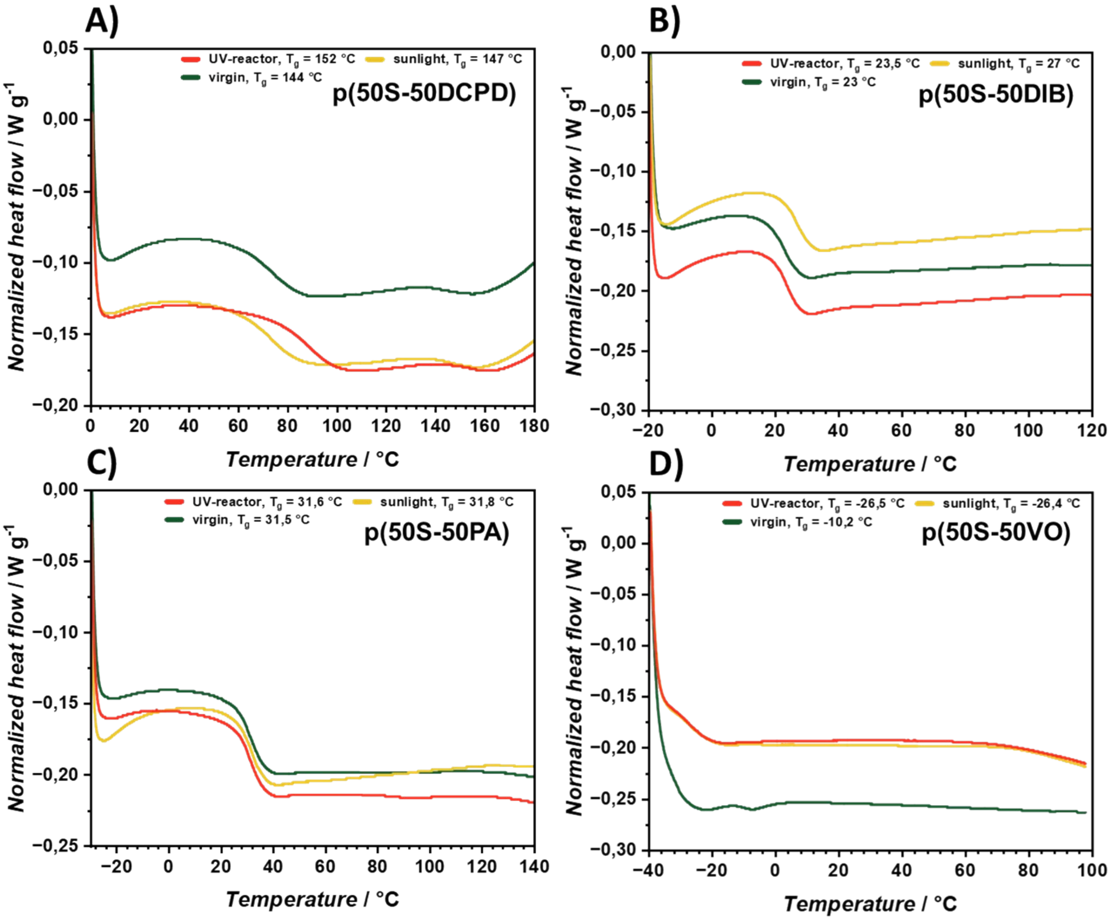

Figure S 8: DSC thermograms of p(50S-50DCPD), p(50S-50DIB), p(50S-50PA) and p(50S-50VO) before and after exposure to sunlight and concomitant temperature variations, as well as UV light (365 nm) in the custom-made photo-reactor.

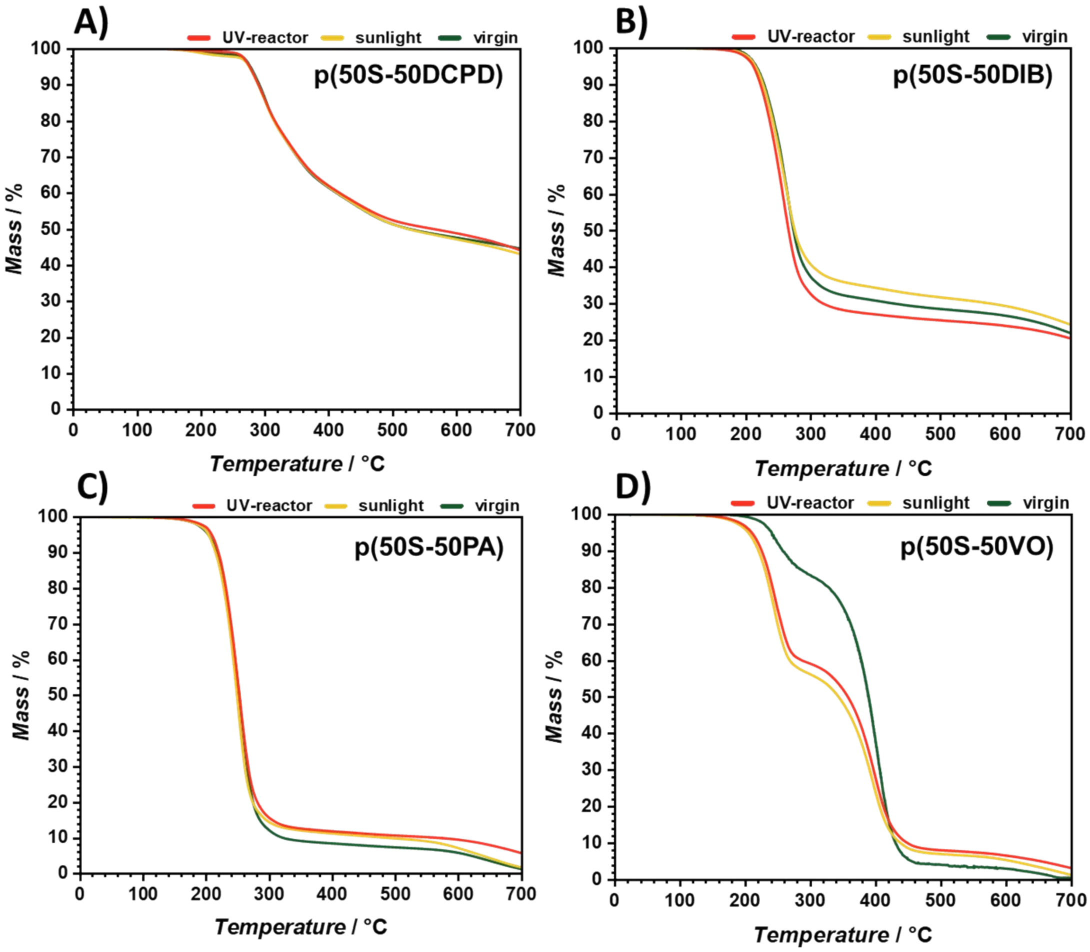

Figure S 9: TGA thermogram of p(50S-50DCPD), p(50S-50DIB), p(50S-50PA) and p(50S-50VO) before and after exposure to sunlight and concomitant temperature variations, as well as UV light (365 nm) in the custom-made photo-reactor.

Water sorption

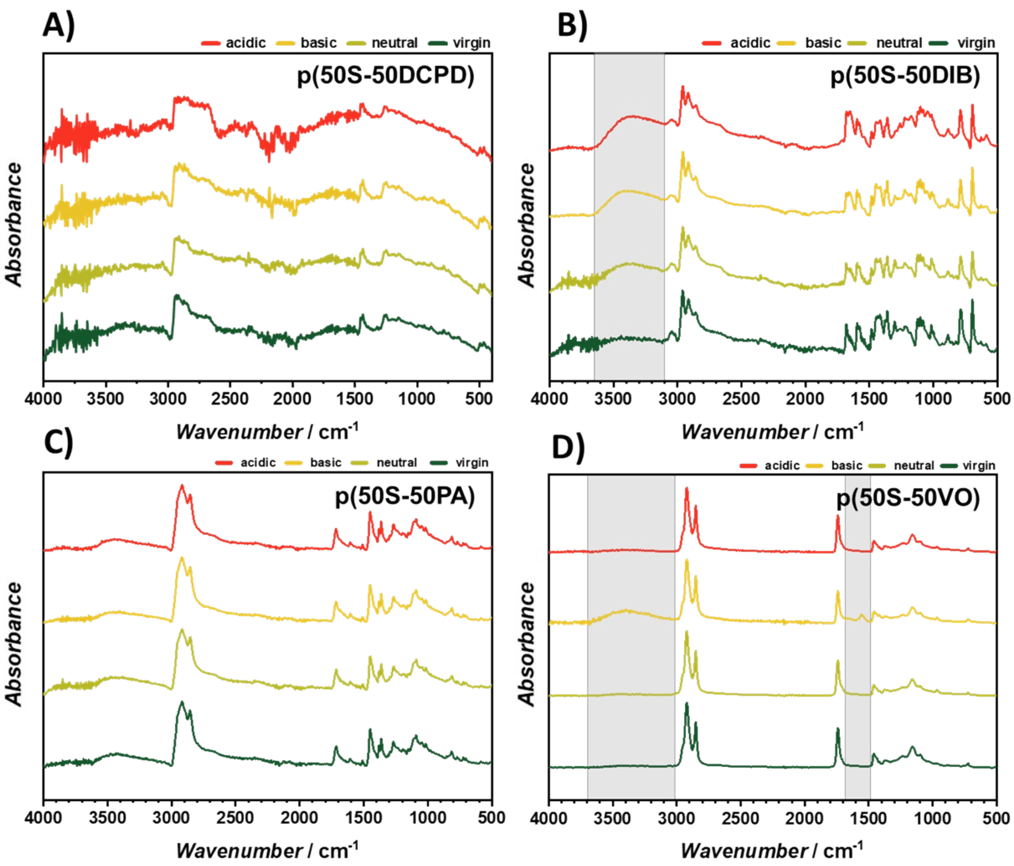

Figure S 10: FTIR spectra of p(50S-50DCPD), p(50S-50DIB), p(50S-50PA) and p(50S-50VO) before and after immersion in deionised water, 0.1 M NaOH and 0.1 M HCl solution for 110 days.

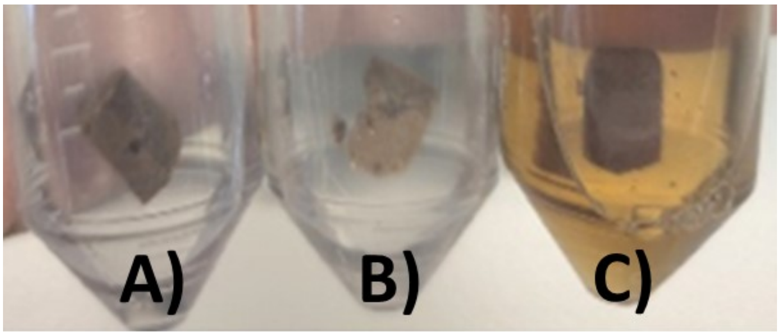

Figure S 11: Image of p(50S-50VO) after immersion in **A)** deionised water, **B)** 0.1 M HCl and **C)** 0.1 M NaOH solution for 110 days, with visible colouration after hydrolysis and leaching under basic conditions.

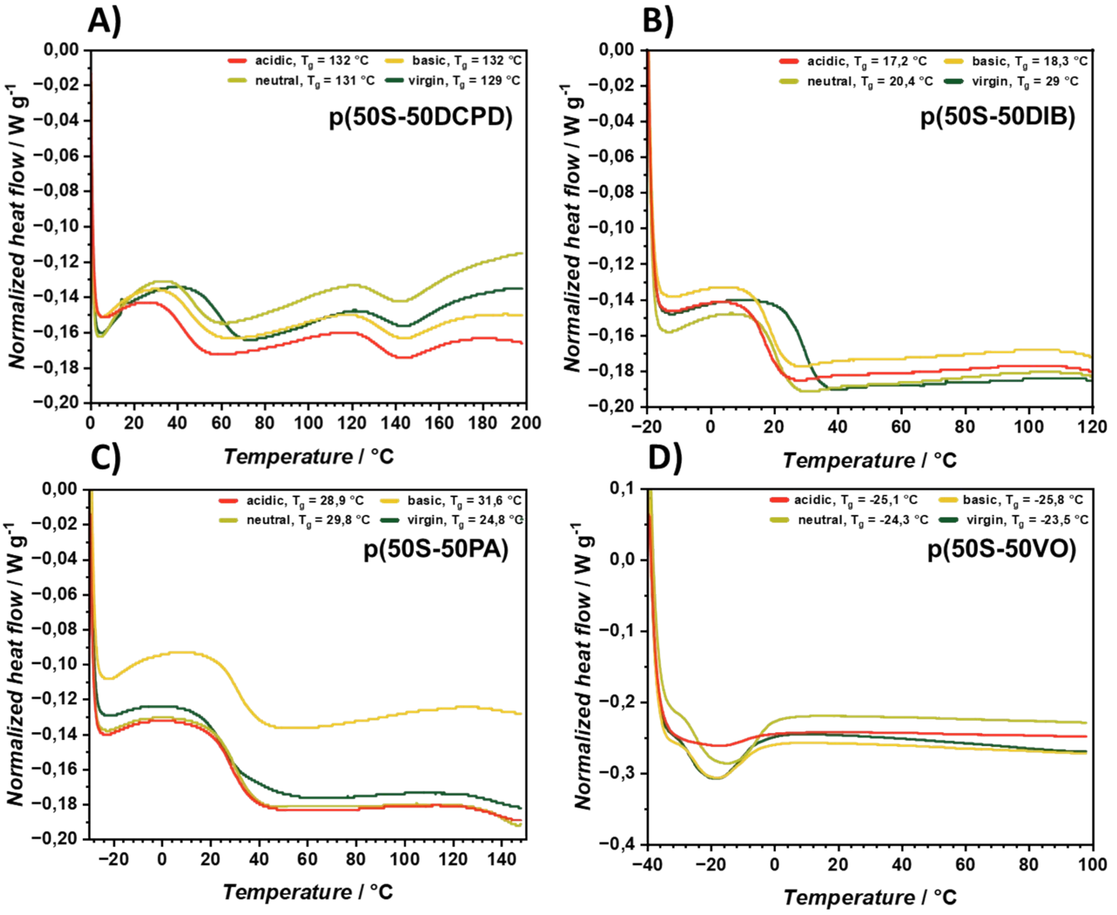

Figure S 12: DSC thermograms of p(50S-50DCPD), p(50S-50DIB), p(50S-50PA) and p(50S-50VO) before and after immersion in deionised water, 0.1 M NaOH and 0.1 M HCl solution for 110 days.

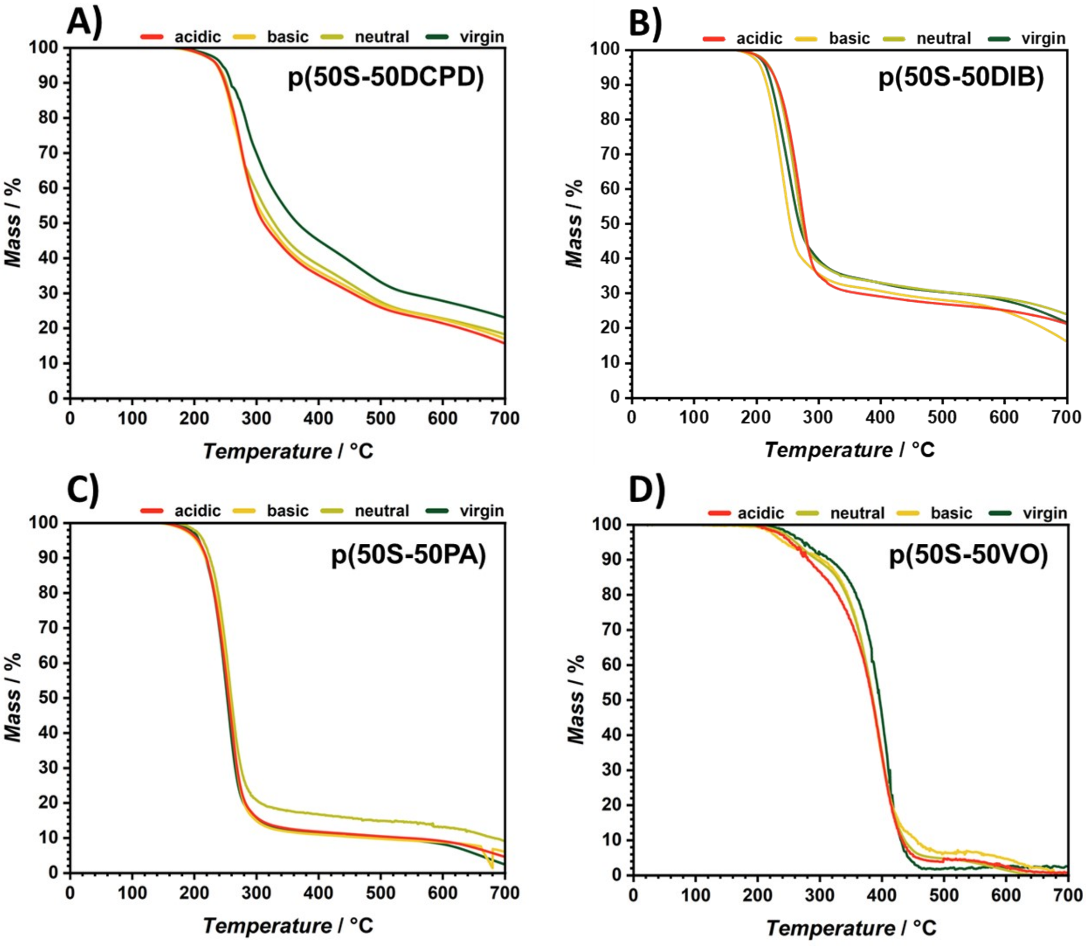

Figure S 13: TGA thermogram of p(50S-50DCPD), p(50S-50DIB), p(50S-50PA) and p(50S-50VO) before and after immersion in deionised water, 0.1 M NaOH and 0.1 M HCl solution for 110 days.

Biodegradation and cytotoxicity

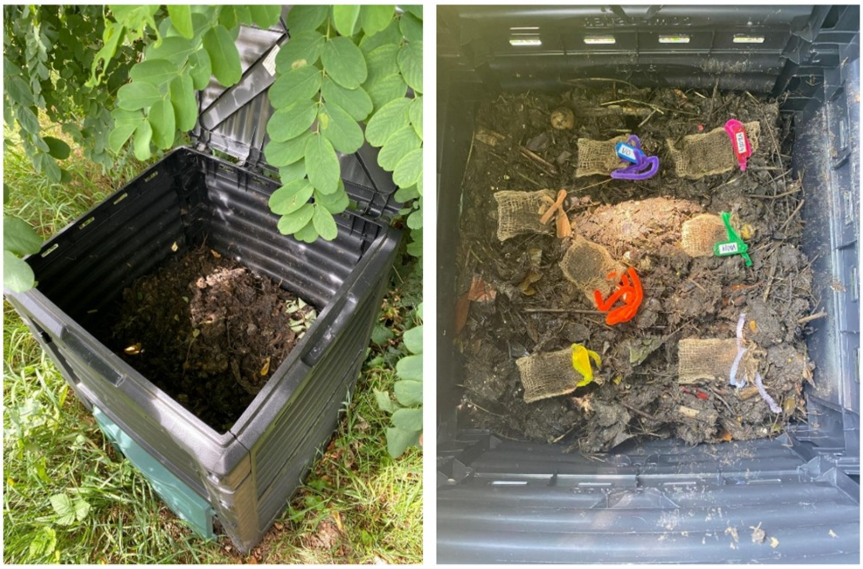

Figure S 14: Image of the household compost used for biodegradation studies.

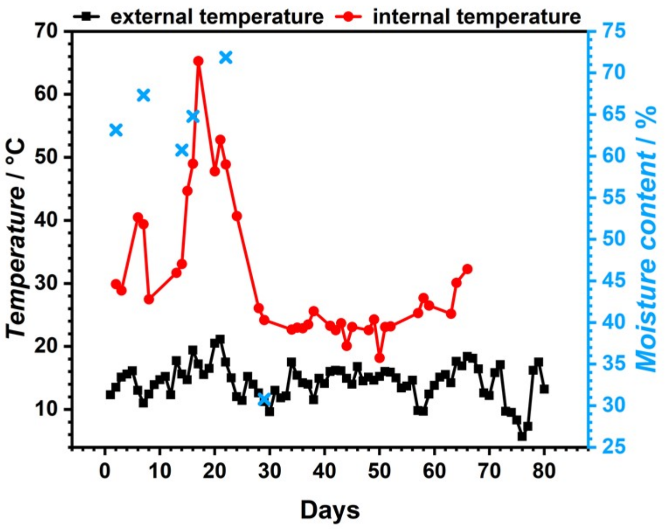

Figure S 15: Environmental and internal composter temperature trend during the biodegradation studies, as well as moisture content during the early stages of the experiment.

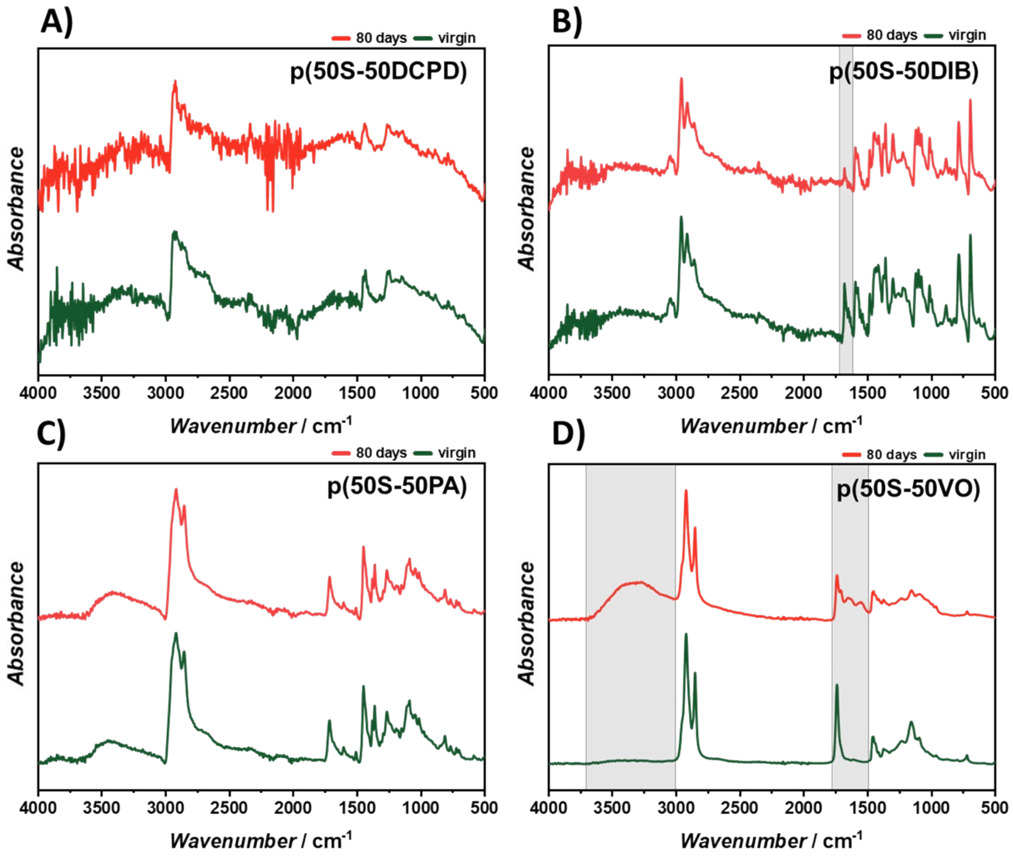

Figure S 16: FTIR spectra of p(50S-50DCPD), p(50S-50DIB), p(50S-50PA) and p(50S-50VO) before and after exposure to biological attack in a household compost for 80 days.

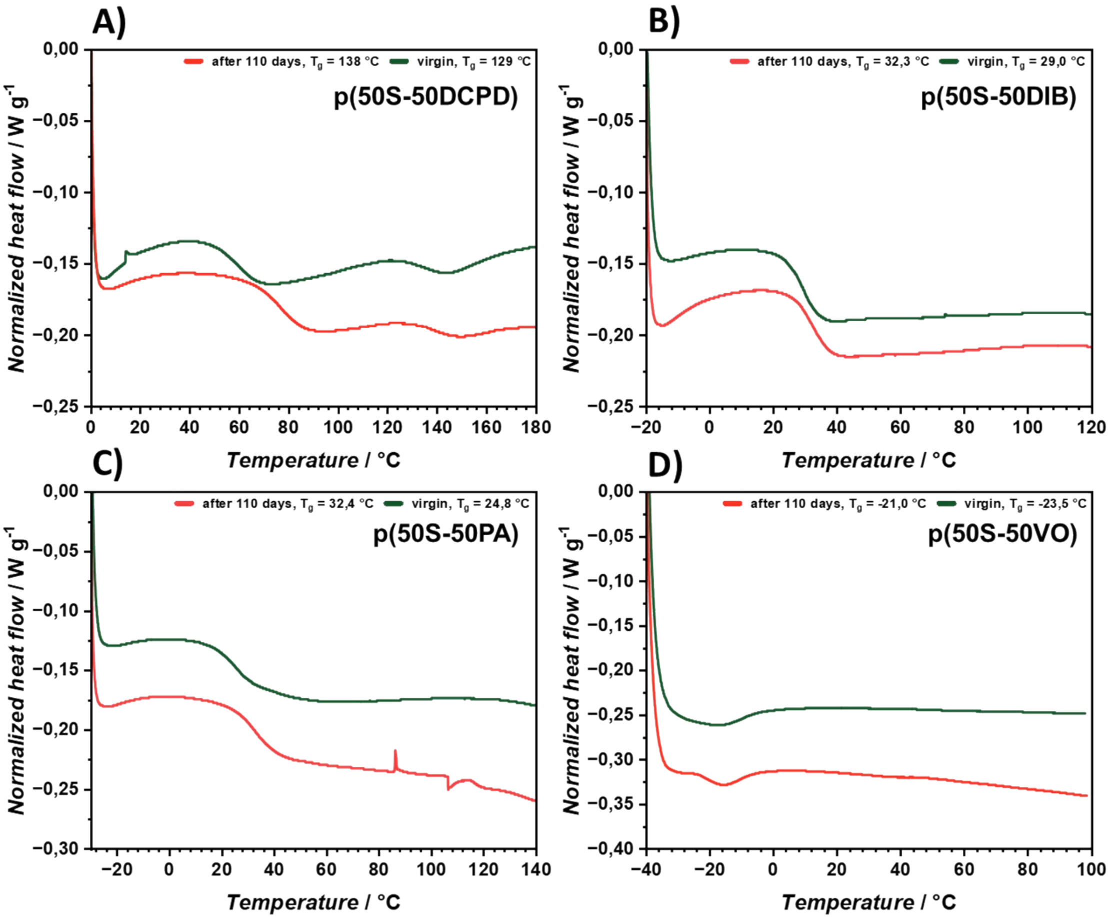

Figure S 17: DSC thermograms of p(50S-50DCPD), p(50S-50DIB), p(50S-50PA) and p(50S-50VO) before and after exposure to biological attack in a household compost for 80 days.

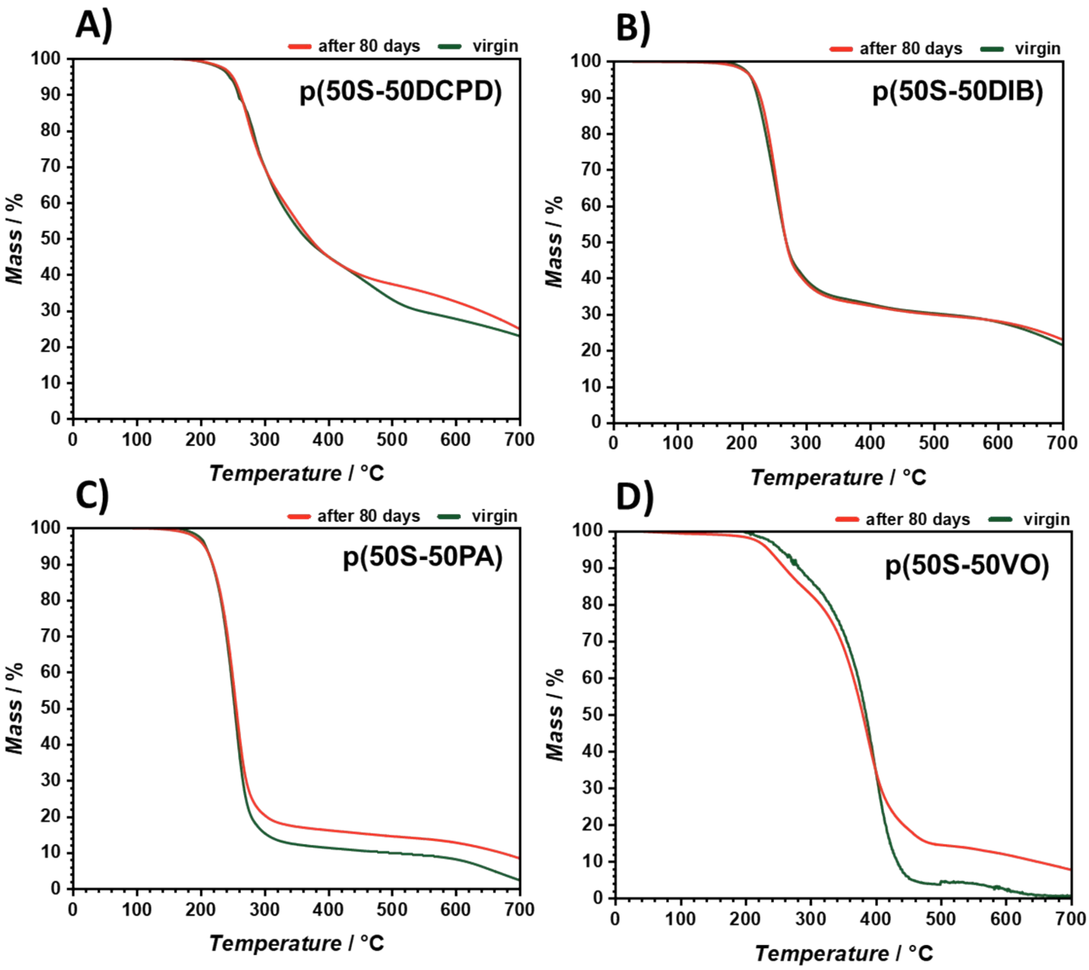

Figure S 18: TGA thermogram of p(50S-50DCPD), p(50S-50DIB), p(50S-50PA) and p(50S-50VO) before and after exposure to biological attack in a household compost for 80 days.

TGA data summary

Table S 1: Summary of all TGA data including the four inverse vulcanised polymer samples with $T_{5\%}$ and wt.% solid residue after thermal degradation.

		p(50S-50DCPD)				p(50S-50DIB)				p(50S-50PA)				p(50S-50VO)			
		Virgin		Aged		Virgin		Aged		Virgin		Aged		Virgin		Aged	
		$T_{5\%}$ [°C]	Residue [wt.%]	$T_{5\%}$ [°C]	Residue [wt.%]	$T_{5\%}$ [°C]	Residue [wt.%]	$T_{5\%}$ [°C]	Residue [wt.%]	$T_{5\%}$ [°C]	Residue [wt.%]	$T_{5\%}$ [°C]	Residue [wt.%]	$T_{5\%}$ [°C]	Residue [wt.%]	$T_{5\%}$ [°C]	Residue [wt.%]
**Thermal ageing**		277	47	283	47	222	25	268	51	203	1	227	15	252	3	294	20
**UV ageing**	**sunlight**	276	44	276	43	215	22	214	25	203	1	203	2	241	<1	210	1
	**UV-reactor**			276	44			211	20			209	6			205	3
**Water sorption**	**neutral**	244	23	237	18	215	22	222	24	207	3	215	9	252	<1	261	<1
	**basic**			237	17			221	16			204	6			243	<1
	**acid**			237	16			207	22			204	5			274	3
**Bio attack**		245	23	250	25	215	22	215	23	207	3	205	9	252	<1	235	8

Proposed ageing mechanics

Here, we propose a series of ageing mechanisms for IV polymers, based on our data, and from our expanded literature search. All shown mechanisms are simplified without possible by-products.

Reduction of sulfur rank

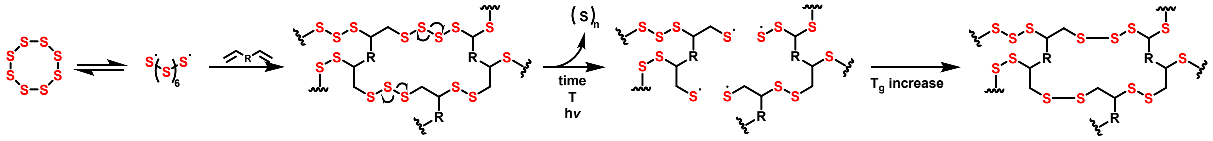

Scheme S 1: Proposed simplified mechanism of decreasing sulfur ranks and concomitant increase of T_g due to exposure to elevated temperatures and radiation.

The sulfur rank (number of sulfur atoms in a chain between organic moieties), is suggested to be a major influence on the physical and thermomechanical properties of inverse vulcanised polymers. A longer sulfur rank may lead to a more flexible polymer, while a shorter sulfur rank would provide a brittle material with high T_g due to the restriction in mobility of the polymer chains. This suggests a liberation of sulfur from within the sulfur chain, with dynamic S-S bond breaking, release of sulfur, and reforming of the S-S bonds to repair the polymer chain.[1-3] Further, It is known that S-S bonds can be dynamic, with or without stimulus. A rheological study conducted by Bischoff et al. suggested living character driven by homolytic S-S bond cleavage, with different IV polymers demonstrating Maxwellian or covalent adaptive network (CAN) behaviour.[4]

Oxidation

p(50S-50DCPD)

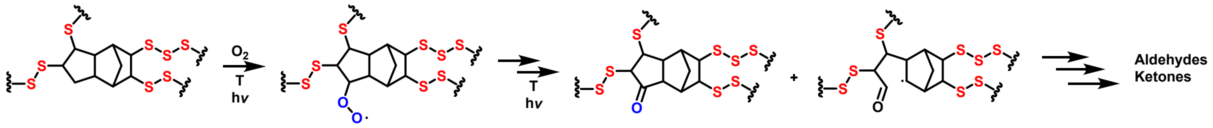

Scheme S 2: Proposed mechanism of oxidative degradation of p(50S-50DCPD) due to exposure to oxygen under elevated temperatures and radiation.[5]

p(50S-50DIB)

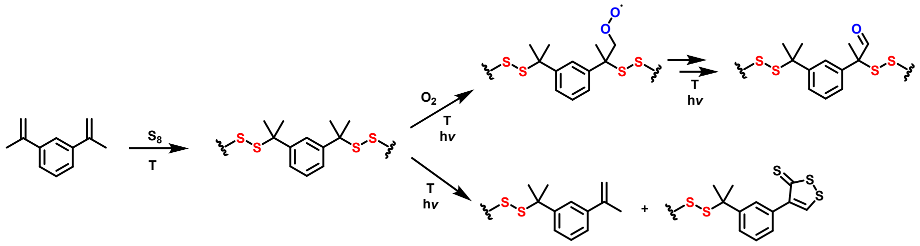

Scheme S 3: Proposed mechanism of degradation of p(50S-50DIB) due to exposure to oxygen and/or elevated temperatures and radiation.[6]

p(50S-50PA)

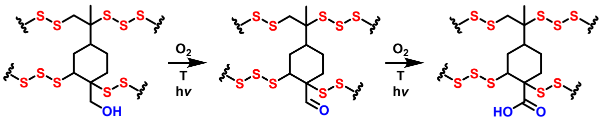

Scheme S 4: Proposed mechanism of oxidative degradation of p(50S-50PA) due to exposure to oxygen under elevated temperatures and radiation.

p(50S-50VO)

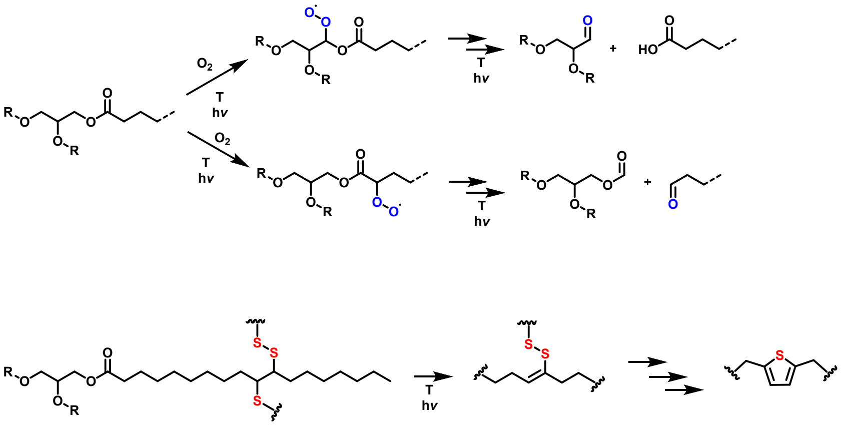

Scheme S 5: Proposed mechanism of degradation of p(50S-50VO) due to exposure to oxygen and/or elevated temperatures and radiation.[7-9]

Hydrolysis

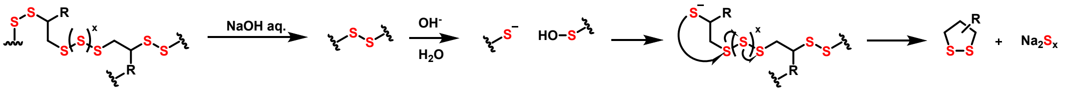

Scheme S 6: Proposed degradation mechanism of organic polysulfides under alkaline condition.[10]

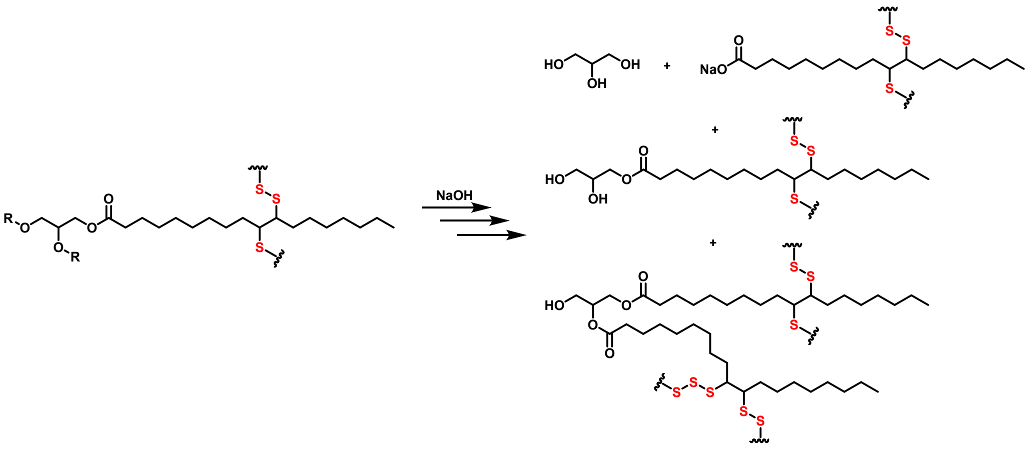

Scheme S 7: Proposed mechanism of hydrolytic degradation of p(50S-50VO) under basic conditions.

References

1 J. J. Dale, S. Petcher, T. Hasell, *ACS Applied Polymer Materials* **2022**, *4*, 3169-3173.
2 J. J. Dale, J. Stanley, R. A. Dop, G. Chronowska-Bojczuk, A. J. Fielding, D. R. Neill, T. Hasell, *European Polymer Journal* **2023**, *195*.
3 J. Dale, Dissertation thesis, University of Liverpool (United Kingdom), **2024**.
4 D. J. Bischoff, T. Lee, K.-S. Kang, J. Molineux, W. O'Neil Parker, J. Pyun, M. E. Mackay, *Nature Communications* **2023**, *14*.
5 J. Huang, W. Minne, R. Drozdzak, G. Recher, P. Y. Le Gac, E. Richaud, *Polymer Degradation and Stability* **2020**, *174*.
6 J. Bao, K. P. Martin, E. Cho, K. S. Kang, R. S. Glass, V. Coropceanu, J. L. Bredas, W. O. Parker, Jr., J. T. Njardarson, J. Pyun, *J Am Chem Soc* **2023**, *145*, 12386-12397.
7 D. W. Johnson, in *Recent Progress in Some Aircraft Technologies* (Ed.: R. K. Agarwal), IntechOpen, **2016**.
8 B. Zheng, L. Zhong, X. Wang, P. Lin, Z. Yang, T. Bai, H. Shen, H. Zhang, *Nat Commun* **2024**, *15*, 5507.
9 G. Gryn'ova, J. L. Hodgson, M. L. Coote, *Org Biomol Chem* **2011**, *9*, 480-490.
10 Y. Deng, Z. Huang, B. L. Feringa, H. Tian, Q. Zhang, D. H. Qu, *Nat Commun* **2024**, *15*, 3855.
